# Fast reconstruction of degenerate populations of conductance-based neuron models from spike times

**DOI:** 10.1371/journal.pcbi.1014337

**Published:** 2026-05-21

**Authors:** Julien Brandoit, Damien Ernst, Guillaume Drion, Arthur Fyon

**Affiliations:** 1 Department of Electrical Engineering and Computer Science, University of Liège, Liège, Belgium; 2 LTCI, Telecom Paris, Institut Polytechnique de Paris, Palaiseau, France; University of Edinburgh, UNITED KINGDOM OF GREAT BRITAIN AND NORTHERN IRELAND

## Abstract

Inferring the biophysical parameters of conductance-based models (CBMs) from experimentally accessible recordings remains a central challenge in computational neuroscience. Spike times are the most widely available data, yet they reveal little about which combinations of ion channel conductances generate the observed activity. This inverse problem is further complicated by neuronal degeneracy, where multiple distinct conductance sets yield similar spiking patterns. We introduce a method that addresses this challenge by combining deep learning with Dynamic Input Conductances (DICs), a theoretical framework that reduces complex CBMs to three interpretable feedback components governing excitability and firing patterns. Our approach first maps spike times directly to DIC densities at threshold using a lightweight neural network that learns a low-dimensional representation of neuronal activity. The predicted DIC values are then used to generate degenerate CBM populations via an iterative compensation algorithm, ensuring compatibility with the intermediate target DICs, and thereby reproducing the corresponding firing patterns, even in high-dimensional models. Applied to two neuronal models, this algorithmic pipeline reconstructs spiking, bursting, and irregular regimes with high accuracy and robustness to variability, including spike trains generated under noisy current injection mimicking physiological stochasticity. It produces diverse degenerate populations within milliseconds on standard hardware, enabling scalable and efficient inference from spike recordings alone. Beyond methodological advances, we provide an open-source software package with a graphical interface that allows experimentalists to generate and explore CBM populations directly from spike trains without requiring programming expertise. Together, this work positions DICs as a practical and interpretable link between experimentally observed activity and mechanistic models. By enabling fast and scalable reconstruction of degenerate populations directly from spike times, our approach provides a powerful way to investigate how neurons exploit conductance variability to achieve reliable computation and provides the foundation for experimental applications that span from neuromodulation studies to real-time model-guided interventions.

## Author summary

Neurons communicate through spikes, and spike timing is a crucial part of neuronal processing. Spike times can be recorded experimentally both intracellularly and extracellularly, and are the main output of state-of-the-art neural probes. On the other hand, neuronal activity is controlled at the molecular level by the currents generated by many different transmembrane proteins called ion channels. Connecting spike timing to ion channel composition remains an arduous task to date. To address this challenge, we developed a method that combines deep learning with a theoretical tool called Dynamic Input Conductances (DICs), which reduce the complexity of ion channel interactions into three interpretable components describing how neurons spike. Our approach uses deep learning to infer DICs directly from spike times and then generates populations of “twin” neuron models that replicate the observed activity while capturing natural variability in membrane channel composition. The method is fast, accurate, and works using only spike recordings. We also provide open-source software with a graphical interface, making it accessible to researchers without programming expertise.

## Introduction

A central objective in both experimental and computational neuroscience is to understand how neurons generate and regulate electrical activity from their ion channels, linking microscopic mechanisms to macroscopic dynamics. Extracellular recordings, particularly spike times, remain the most widely accessible data, especially when probing large networks [1–3]. Spike times provide rich information about neural dynamics across brain regions and conditions. However, while they reveal what neurons do and how they respond to external perturbations, they rarely reveal the underlying mechanisms, specifically which combinations of biophysical parameters produce the observed activity. This question is crucial because such biophysical parameters are, among others, targets of neuromodulators and neuroactive medications [4–6].

A major obstacle in linking neuronal activity with biophysical properties is *neuronal degeneracy*, the well-established fact that multiple distinct parameter sets, particularly effective ion channel densities, can produce similar spiking behaviors [7]. This functional flexibility enhances robustness but complicates interpretation, as neurons exhibit inherently nonlinear, high-dimensional dynamics. When different parameter combinations produce indistinguishable activity, identifying which configurations underlie recorded behavior becomes an underdetermined problem [8–10].

Conductance-based models (CBMs) provide a principled framework to tackle this challenge computationally. Their parameters, such as maximal ion conductances, link directly to measurable biological properties like ion channel expression. However, realistic CBMs are complex, often requiring dozens of parameters [9,11–13]. Combined with degeneracy, this complexity makes inferring conductances from experimental data highly challenging and often computationally intractable, with results that are difficult to interpret.

This difficulty defines a core inverse problem in computational neuroscience: *given an observed activity pattern such as a spike train, how can one identify a set (or better, the population) of biophysical models that could have generated it?* Existing approaches face key limitations. Many require full voltage traces or intracellular recordings, making them sensitive to noise and less feasible in practice [14–22]. Recent advances in simulation-based inference (SBI) have partially addressed these limitations by training neural density estimators to approximate posterior distributions over parameters given observed data, enabling fast inference while capturing parameter uncertainty [23–28]. Related efforts using data assimilation and variational methods have a long history in this context, notably the work of Abarbanel and collaborators on dynamical state and parameter estimation in conductance-based models [29,30], as well as subsequent applications to automated model construction [31]. However, these approaches operate directly in the high-dimensional conductance space without explicit reference to the dynamical mechanisms that shape neuronal activity. As a result, the learned posteriors, though valid, do not straightforwardly reveal *why* certain parameter combinations produce similar outputs, and the relationship between parameter variability and functional equivalence remains implicit.

In this work, we introduce a method that addresses this inverse problem by combining deep learning with Dynamic Input Conductances (DICs) [32,33], a theoretical framework from dynamical systems theory that reduces complex CBMs to three interpretable feedback components governing excitability and firing patterns. Our approach uses DICs as a low-dimensional intermediate: a lightweight deep learning architecture first maps spike times to DIC values at threshold, and an iterative compensation algorithm then generates degenerate CBM populations compatible with these values. Given only spike times, the method outputs a population of degenerate CBMs within milliseconds on standard hardware.

We validate the pipeline on two distinct models and show that it faithfully reconstructs neuronal activity across spiking, bursting, and irregular regimes, while maintaining robustness to variability and noise. By combining deep learning with DIC theory, this approach provides a practical solution to the inverse inference problem. It bridges experimentally accessible observables and mechanistic CBMs, demonstrates that DICs serve as interpretable low-dimensional intermediates, and enables scalable reconstruction of degenerate populations from spike times alone.

To support widespread adoption and facilitate daily use in experimental settings, we provide our entire pipeline as an open-source software package with a graphical interface [34]. Recognizing that many researchers in the biomedical sciences come from diverse backgrounds where programming and numerical modeling are not core skills, the tool is designed to be intuitive and easy to use, allowing experimentalists to generate and explore model populations directly from spike recordings without writing code.

## Results

### General problem statement

In this work, we assumed a known conductance-based model (CBM) whose membrane dynamics is described by:


$$\begin{array}{c} C\frac{dV}{dt}+g_{\text{leak}}(V-E_{\text{leak}})=-\sum_{i\in ℐ}{\bar{g}}_{i}m_{i}^{p_{i}}h_{i}^{q_{i}}(V-E_{i})+I_{\text{ext}}, \end{array}$$

where *V* denotes the membrane potential, *C* is the membrane capacitance, *g*_leak_ and *E*_leak_ are the leak conductance and reversal potential, and each ionic current i∈ℐ is characterized by its maximal conductance ${\bar{g}}_{i}$, gating variables *m*_*i*_ and *h*_*i*_ with powers *p*_*i*_ and *q*_*i*_, and reversal potential *E*_*i*_. The external current input is denoted by *I*_ext_. In this formulation, the structure of the model (i.e., the functional form of the ionic currents and their gating dynamics) was assumed to be fixed and known (see Materials and Methods). The unknown parameters are the conductances $\bar{g}=[{\bar{g}}_{1},\ldots ,{\bar{g}}_{\left|ℐ\right|},g_{\text{leak}}]\in 𝒢$, which must be inferred from observations extracted from the voltage trace. A specific choice of $\bar{g}$ fully specifies one instance of the considered CBM, while all other parameters (e.g., reversal potentials, capacitance, …) are assumed to be known and fixed; inferring these remains an avenue for future work. We chose spike times as representation of the activity, as these data are easily accessible from both intracellular and extracellular recordings. We denote such a recorded spike times sequence by:


$$\begin{array}{c} x=[t_{1},t_{2},\ldots ,t_{N_{\text{spikes}}}]. \end{array}$$

The quantity *N*_spikes_ denotes the total number of spikes detected in the recording. Its value depends both on the duration of the recording and on the firing activity of the neuron, which makes *x* a variable-length representation of the neuronal activity.

Due to degeneracy [4,35], the mapping:


$$\begin{array}{c} x⟼\bar{g}\in {𝒢}^{*}(x), \end{array}$$

is not bijective: the solution to the inference problem is not a single point, but rather a subspace ${𝒢}^{*}(x)\subset 𝒢$ containing infinitely many parameter sets compatible with *x* [26,32,33,36].

Our objective is to build a set of *P* models (with *P* freely chosen by the experimentalist) with different conductance values that all reproduce a firing pattern similar to *x*. We call this set 𝒫, and since each model is defined by its value of $\bar{g}$ we write $𝒫={\left\{[{\bar{g}}_{1},\ldots ,{\bar{g}}_{\left|ℐ\right|},g_{\text{leak}}{]}_{i}\right\}}_{i=1}^{P}$. We can build such a set by generating *P* instances from the subspace ${𝒢}^{*}(x)$.

While existing methods either infer a single solution or attempt to learn the full high-dimensional solution space directly (see Introduction), we adopt an intermediate strategy. This strategy divides the problem into two parts and leverages intermediate low-dimensional representations of CBMs called DICs (see Materials and Methods) [32,33,37,38]. For any CBM, we can analytically construct a DIC representation determined by:


$$\begin{array}{c} g_{\text{DICs}}(V)=S(V;\bar{g})\cdot \bar{g}, \end{array}$$ (1)

where $S=\frac{\partial g_{\text{DICs}}}{\partial \bar{g}},$ is the sensitivity matrix determined by the CBM as in [33], and $g_{\text{DICs}}(V):V\mapsto {\mathbb{R}}^{3}$ provides a low-dimensional equivalent of the high-dimensional conductance vector $\bar{g}$. Importantly, the DIC curves evaluated at a specific potential, the threshold potential *V*_th_, are sufficient to capture most of the spontaneous activity associated with a given CBM [32,33]. The threshold potential corresponds to the voltage at which the neuron is maximally sensitive to changes in its conductance parameters (see Materials and Methods).

The three components of the DIC representation correspond to distinct timescales of the membrane dynamics, giving rise to three separate DIC curves: the fast component *g*_f_(*V*), the slow component *g*_s_(*V*), and the ultra-slow component *g*_u_(*V*). Each curve captures the influence of ionic conductances acting on its respective timescale. Because these curves evaluated at a single characteristic voltage are sufficient to capture most of the spontaneous activity (Eq. 1), the full conductance space effectively reduces to just three scalars, providing a compact summary of all conductance configurations that produce similar firing activity.

**Fig 1:**
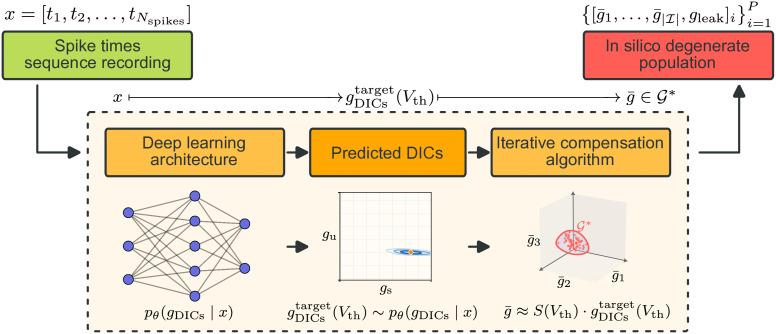
Our proposed approach. Spike time sequences are processed by a deep learning model that predicts DICs, a compact representation of the high-dimensional conductance space. These predicted DIC values serve as targets for an iterative compensation algorithm, which explores the degenerate solution space to generate multiple conductance configurations $\bar{g}$ that reproduce the input spike pattern. This two-step strategy (i) reduces the dimensionality of the inference problem, and (ii) leverages compensation to recover diverse biologically plausible parameter sets consistent with the observed activity.

Leveraging this intermediate representation, our approach proceeds in two steps (Fig 1). First, a *deep learning architecture* learns the mapping from observed spike times *x* to the corresponding DIC values. Because different activity regimes are governed by a different number of timescales, degeneracy can also arise within the DIC space itself, particularly for spiking activity; we therefore perform posterior density estimation and sample from the learned density to obtain $g_{\text{DICs}}^{\mathrm{target}}(V_{\text{th}})$. This enables parameter inference in a tractable, low-dimensional space by inferring the left-hand side of relation (1). Second, given a predicted DIC vector, we generate valid CBM conductance vectors $\bar{g}$ that are compatible with these DIC values, thus recovering diverse, biologically plausible instances of the original high-dimensional model. For this step, we build upon the compensation method introduced in [33], which generates degenerate CBM populations from DIC constraints, employing an iterative extension that improves constraint satisfaction in models with nonlinear compensatory structure (see Materials and Methods). Overall, our approach reads:


$$\begin{array}{c} x\overset{\text{Deep learning architecture}\;}{\underset{\;\;}{\to }}g_{\text{DICs}}^{\mathrm{target}}(V_{\text{th}})\overset{\text{Compensation algorithm}\;}{\underset{\;\;}{\to }}\bar{g}\in {𝒢}^{*}. \end{array}$$

This work addresses three components: (i) building a synthetic dataset linking DIC values to a diverse range of neuronal activities; (ii) designing a deep learning architecture capable of processing variable-length spike time sequences and predicting plausible DIC targets; and (iii) validating the complete pipeline, from spike times to degenerate populations, across models and under realistic noise conditions.

### DICs provide a structured and learnable representation of neuronal activity

To train the deep learning architecture and characterize the relationship between DIC values and neuronal activity, we constructed a large synthetic dataset spanning a broad range of DIC values and corresponding spike trains [39]. Rather than sampling conductance parameters directly, we uniformly sampled the slow and ultra-slow DICs $g^{*}=\left(g_{\text{s}}(V_{\text{th}});\;g_{\text{u}}(V_{\text{th}})\right)$ across a wide bounded region, and generated for each sampled pair a degenerate population of CBM instances using the iterative compensation algorithm. Each instance was simulated under noisy current injection mimicking physiological stochasticity, yielding a total of |𝒯|=1,200,000 neurons spanning spiking, bursting, and irregular regimes. Full details of the dataset construction are provided in the Materials and Methods.

**Fig 2:**
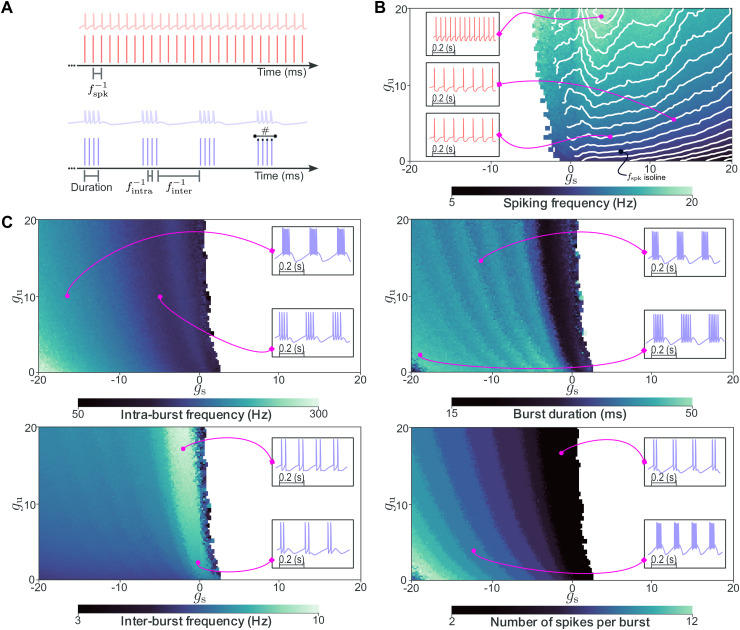
Activity descriptors vary smoothly across the DIC space. Heatmaps show how individual activity metrics vary across the DIC space, revealing clear gradients that reflect a nonlinear yet structured relationship between DIC constraints and neuronal firing patterns. **(A)** Representative example traces of a spiking neuron (top) and a bursting neuron (bottom), with annotations highlighting the descriptors extracted from each regime: mean spiking frequency for the spiking case, and mean burst duration, mean intra-burst frequency, mean inter-burst frequency, and mean number of spikes per burst for the bursting case. **(B)** Spiking metrics summarized by mean firing rate. **(C)** Bursting metrics including intra-burst frequency, inter-burst frequency, burst duration, and spikes per burst. These smooth gradients support the learnability of the inverse mapping from spike trains to DICs, and position DICs as meaningful low-dimensional intermediates for characterizing neuronal activity.

This dataset reveals a clear and structured relationship between DIC values and neuronal firing patterns (Fig 2). Each activity descriptor can be viewed as a function of the sampled slow and ultra-slow DICs. A sharp separation between spiking and bursting neurons emerges, with a transition largely independent of *g*_u_ and located near $g_{\text{s}}\approx 0$, consistent with prior work [32,33]. Negative slow DIC values at threshold are associated with bursting, while positive values lead to spiking. The overlapping zone at the transition is due to heterogeneous populations. Outside this narrow transition zone, generated populations are fully homogeneous: all instances exhibit the nominal activity type (spiking or bursting) with no failures, as quantified by the entropy measure reported in the Supporting Information (see S1 Appendix).

Beyond this qualitative separation, smooth gradients appear across the DIC space for both bursting descriptors (Fig 2A) and spiking frequency (Fig 2B), revealing a structured and nonlinear relationship between DIC targets and resulting activity. The ranges of the colorbars confirm that the dataset spans a wide variety of activity descriptors (similar to those reported in [15]). Crucially, different neuronal activities are characterized by different DIC values, yet multiple distinct DIC configurations can map to the same activity regime, especially in spiking. This many-to-one correspondence reflects the intrinsic degeneracy of neuronal systems and the timescale separation between activity regimes.

This is the first dataset to systematically map descriptive activity metrics across the DIC space. The smoothness of these gradients is particularly important: it implies that small changes in DIC values correspond to gradual changes in firing behavior, which supports the learnability of the inverse mapping from spike times to DICs by a neural network. Together, these patterns establish DICs as a practical, low-dimensional, and interpretable intermediate that quantitatively links conductance configurations to experimentally observable firing patterns.

The utility of DICs as a learnable intermediate, however, depends on the ability to map predicted DIC values back to conductance vectors $\bar{g}$. This inverse step exploits the relationship $g_{\text{DICs}}(V_{\text{th}})=S(V_{\text{th}};\bar{g})\cdot \bar{g}$ between DICs and maximal conductances. By partitioning $\bar{g}$ into a randomly sampled subset ${\bar{g}}_{\text{random}}$ and a compensated subset ${\bar{g}}_{\text{comp.}}$, the system can be solved for ${\bar{g}}_{\text{comp.}}$ given a target DIC vector, while different draws of ${\bar{g}}_{\text{random}}$ yield different valid solutions, thereby generating a degenerate population as in [33,38]. We emphasize that the partition is exhaustive: together, the two subsets contain all components of $\bar{g}$. This single-step linear compensation is exact when the sensitivity matrix *S* does not depend on the compensated conductances. However, in models where internal dynamics (such as intracellular calcium concentration in the STG model) introduce nonlinear dependencies, the linear approximation can leave residual errors between target and enforced DIC values. To address this, we employ an iterative extension in which the sensitivity matrix is recomputed at each iteration based on the current estimate of ${\bar{g}}_{\text{comp.}}$, progressively reducing these residuals (see Materials and Methods). In practice, five iterations suffice to reduce residual norms by approximately a factor of 15 compared to the single-step method, yielding tighter distributions of activity statistics across generated populations while preserving degeneracy (see Supporting Information, S1 Appendix). This compensation step is used both for generating the synthetic training dataset and as an integral part of the full pipeline at inference time.

### A deep learning architecture efficiently encodes spike trains into DIC targets

The first block of our pipeline (Fig 1) is a deep learning architecture that maps raw spike time sequences directly to a density over DIC values at threshold, without relying on hand-crafted summary statistics. From this learned density, a plausible DIC target is sampled and passed to the iterative compensation algorithm. The architecture and training procedure are described in detail in the Materials and Methods.

To assess whether the learned latent representation captures biologically meaningful structure, we evaluated two auxiliary prediction heads operating solely on the latent embedding: a classification head predicting firing regime (spiking or bursting), and a regression head predicting key activity descriptors. To isolate intrinsic properties from input-driven variability, these metrics were computed on an equivalent dataset simulated without injected current, such that activity descriptors reflect solely the intrinsic conductance properties of each neuron, free from the additional variability introduced by injected stochastic inputs. The model achieved 99.83% classification accuracy and accurately predicted mean spike frequency, intra- and inter-burst frequencies, burst duration, and number of spikes per burst, with mean absolute errors substantially lower than the intrinsic variability of the dataset (Table 1). This confirms that the encoder captures task-relevant temporal structure from raw spike trains.

**Table 1:** Auxiliary regression performance on the STG neuron dataset. Mean absolute error (MAE) values for auxiliary regression tasks are substantially lower than the inherent dataset variability (standard deviation). Descriptors include mean spike frequency (*f*_spk_), intra- and inter-burst frequencies (*f*_intra_, *f*_inter_), burst duration, and mean number of spikes per burst (#).

Descriptor	*f*_spk_	*f*_intra_	*f*_inter_	Duration	Spikes per burst (#)
MAE	0.11 Hz	2.26 Hz	0.15 Hz	0.81 ms	0.16
Dataset std	3.02 Hz	47.91 Hz	1.11 Hz	7.85 ms	2.25

Because degeneracy manifests within the DIC space itself, particularly in the spiking regime where multiple DIC configurations yield functionally indistinguishable activity, pointwise error metrics on predicted DIC values are not a meaningful proxy for pipeline performance. The ultimate criterion is whether the generated populations faithfully reproduce the activity of the input spike trains, which we assess in the following section through a systematic comparison of activity descriptors between input and generated populations. We provide in S1 Appendix evidence that the learned posterior densities are well calibrated following recommendations from [40], demonstrating that the architecture correctly captures degeneracy in the DIC space across both spiking and bursting regimes.

Inference is highly efficient, and, combined with the iterative compensation algorithm, the full pipeline produces degenerate CBM populations within milliseconds on standard hardware.

### The complete generative pipeline reconstructs accurate and diverse degenerate populations from spike times

The complete pipeline combines the deep learning architecture with the iterative compensation algorithm to transform spike time sequences into *in silico* degenerate populations of CBMs, using DICs at threshold as intermediates (Fig 1). This approach generates populations rather than single solutions, capturing a diverse set of conductance combinations compatible with the input activity.

Concretely, given a spike train, the deep learning architecture encodes its temporal structure and yields a density over DIC values compatible with the observed firing pattern, from which one plausible DIC target is sampled. This target is then provided to the iterative compensation algorithm, which generates a set of maximal conductance configurations satisfying the DIC constraints. Because all generated conductance sets are anchored to the same DIC target, they are by construction associated with activity that reproduces the input firing pattern, while spanning a broad and diverse region of the conductance space.

**Fig 3:**
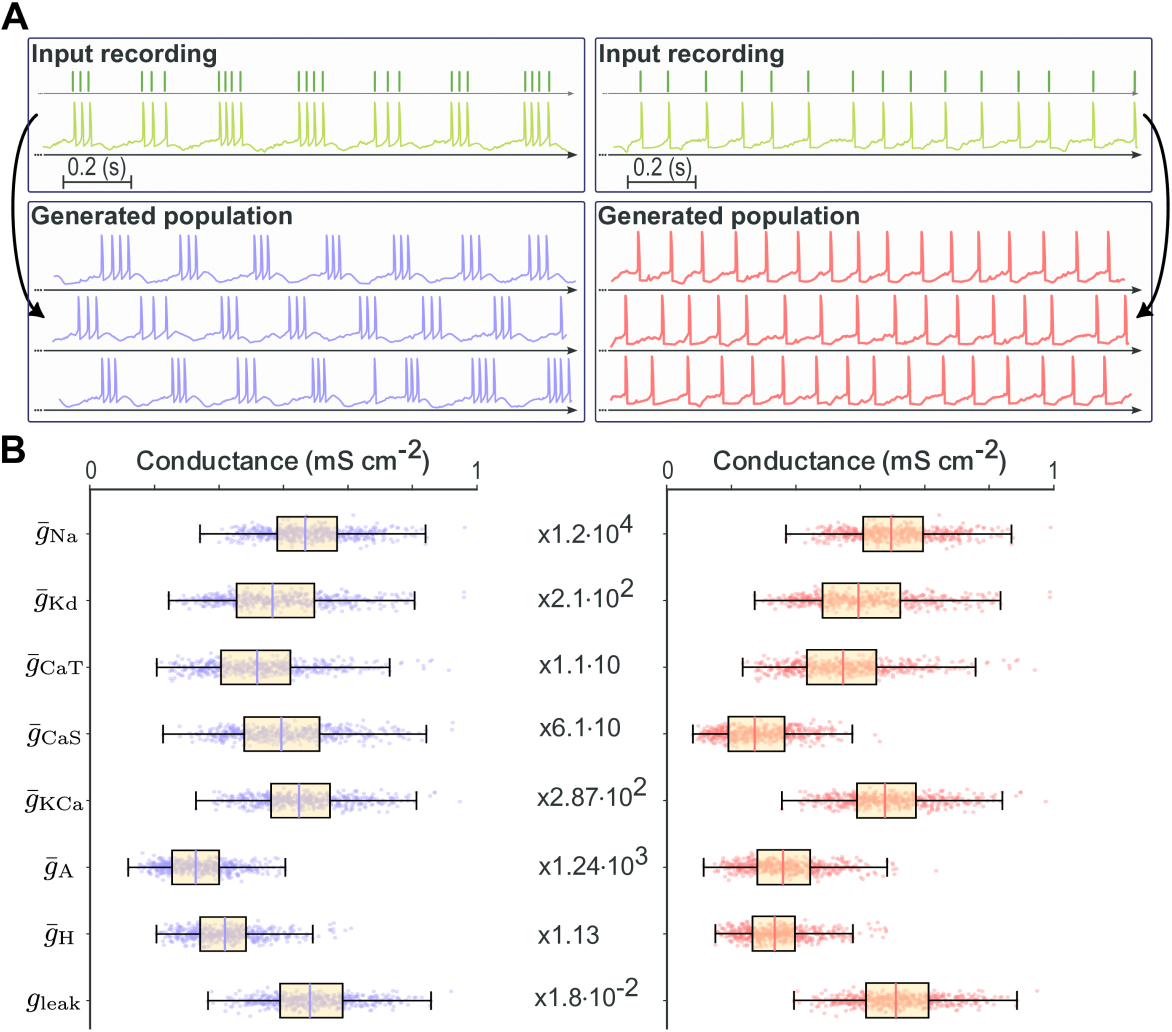
Backbone pipeline output for the STG neuron model. **(A)** Target spike trains (dark green, top) compared with three representative generated spike trains for spiking (red, right) and bursting (purple, left) regimes, showing accurate reproduction of activity patterns. **(B)** Distributions of maximal conductances across 500 generated neurons in spiking (red, right) and bursting (purple, left) regimes, demonstrating broad parameter variability despite similar dynamics.

Qualitative inspection confirms close agreement between generated and target activity patterns in both spiking and bursting regimes (Fig 3A). For each regime, a representative input spike train (dark green) is shown alongside three generated traces, illustrating that the pipeline produces outputs whose temporal structure closely matches the input, with variability confined to levels expected under degeneracy and noisy injected current. Importantly, the method operates solely on spike time input, without requiring voltage traces. However, because the method is blind to subthreshold dynamics, certain features such as after-depolarizations shaping the interspike interval cannot be reproduced.

Across 500 generated neurons per regime, maximal conductance distributions are broad yet yield equivalent spiking or bursting dynamics (Fig 3B), confirming that the pipeline reconstructs multiple distinct solutions producing similar functional outputs, consistent with population-level degeneracy.

**Fig 4:**
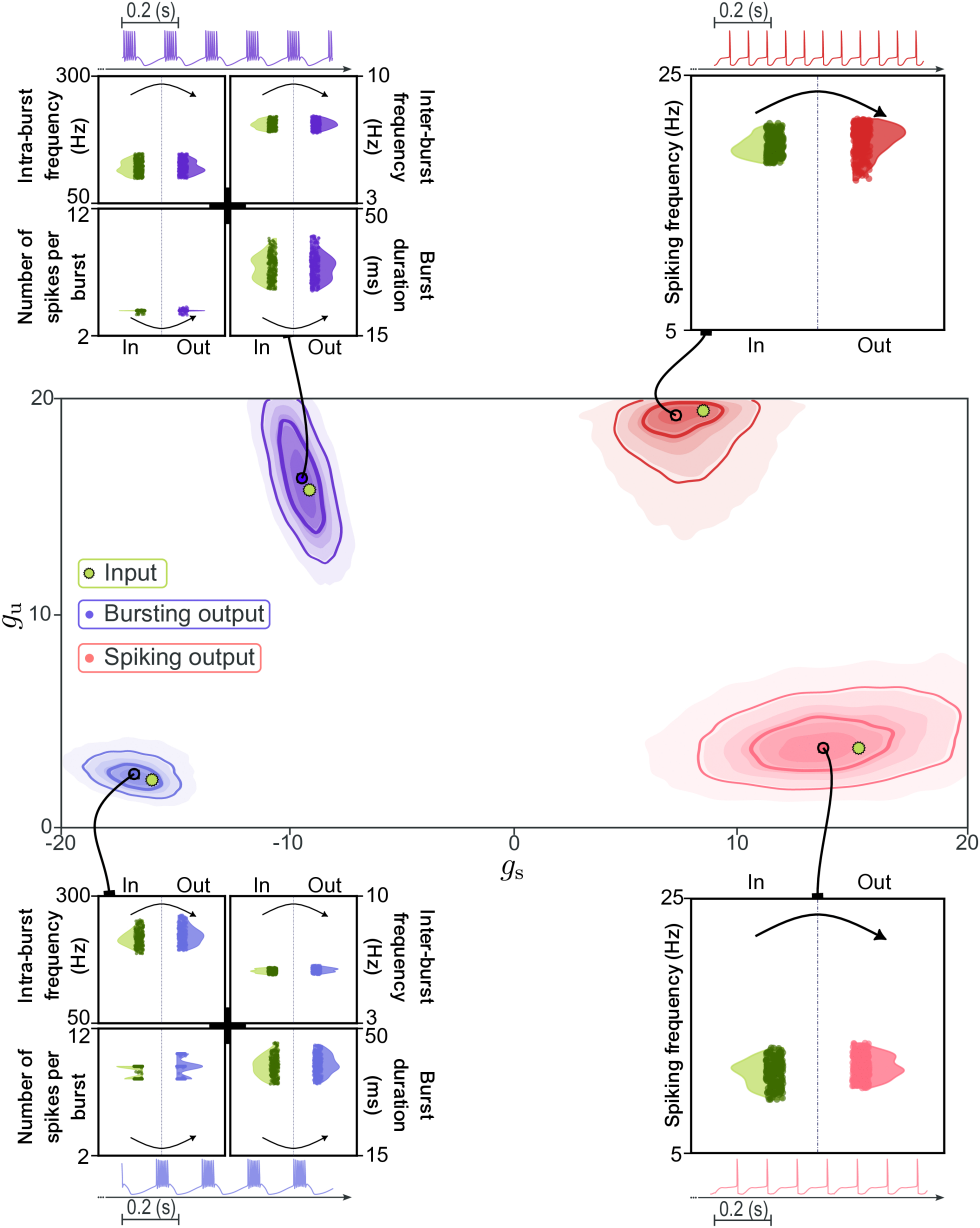
Quantitative comparison of input and generated populations. Four target DIC values (green dots) are selected in the $\left(g_{\text{s}},g_{\text{u}}\right)$ plane, spanning spiking and bursting regimes. For each target, an input population of 256 degenerate neurons is generated via iterative compensation. Spike times from each input neuron are passed through the full pipeline: the deep learning architecture infers a conditional density over DICs (shown as contour plots), from which one pair of DIC targets is sampled and used to generate one neuron each via iterative compensation. Violin plots compare distributions of key activity metrics between the input population (left, green) and the output population (right, colored) for each target point, demonstrating faithful reproduction of firing statistics across regimes.

To quantitatively evaluate reconstruction accuracy, we designed an end-to-end test that mirrors how the pipeline could be used in practice (Fig 4). We selected four target DIC values in the $\left(g_{\text{s}},g_{\text{u}}\right)$ plane, spanning both the spiking and bursting regimes. For each target, we generated an *input population* of 256 neurons using the iterative compensation algorithm. These neurons share identical DIC values at threshold and are therefore degenerate by construction, but still display small variability in their firing patterns arising from three sources: the approximation inherent in constraining DICs only at threshold, the iterative compensation procedure, and the noisy injected current. Such variability is compatible with degeneracy and reflects biological heterogeneity. This first step would, in practice, be replaced by an experimental recording of the activity of a neuron.

We then passed the spike times of each neuron in the input population through the full pipeline. For each input spike train, the deep learning architecture produces a conditional density over DIC values, from which one pair of plausible DIC targets is sampled. Each sampled DIC target is in turn used by the iterative compensation algorithm to generate one new neuron, yielding a total of 256 neurons per target point, which collectively form the *output population*.

Fig 4 shows the inferred DIC densities for each of the four target points, alongside violin plots comparing key activity descriptors between input and output populations. In the bursting regime, the inferred densities are concentrated around the target DIC values, and the output populations closely reproduce the input distributions of intra-burst frequency, inter-burst frequency, burst duration, and number of spikes per burst for both input examples. In the spiking regime, the inferred densities spread more broadly compared to bursting ones, due to the one-dimensional manifold identified earlier (Fig 2B), reflecting the intrinsic DIC degeneracy of spiking activity where multiple $\left(g_{\text{s}},g_{\text{u}}\right)$ combinations yield similar firing rates. Despite this spread in DIC space, the output spiking frequency distributions remain well matched to the inputs, confirming that the pipeline correctly handles this ambiguity by sampling from a region of DIC space that is functionally equivalent. Overall, these results demonstrate that the pipeline faithfully recovers activity features across regimes while preserving degeneracy.

### The pipeline extends to new conductance-based models with minimal retraining

To demonstrate the scalability of our pipeline across distinct CBMs, we applied low-rank adaptation (LoRA) [41] to transfer the backbone model trained on the STG dataset to a dopaminergic (DA) neuron model (see Materials and Methods). This setting highlights a drastic change of context: from a bursting neuron of the stomatogastric ganglion, embedded in the gastric mill rhythm, to a slow pacemaker DA neuron with a completely different conductance composition and dynamical repertoire. LoRA allows the STG-trained backbone to remain intact for its original task, while efficiently adapting to the DA model with a minimal number of additional parameters. By leveraging LoRA, we introduce only approximately 40% of the total number of required parameters for full retraining, reducing both storage and computational demands.

The DA model exhibits three distinct activity regimes: slow pacemaking, fast spiking (a burst that fails to repolarize), and bursting. In the following, we focus on the slow pacemaking and bursting regimes. Despite the marked differences between STG and DA neurons in terms of conductance composition, timescales, and dynamical repertoire, the adapted pipeline successfully handles both tested regimes. This demonstrates that the same organizational principles observed in the STG DIC space, namely clear separation between activity classes and the emergence of manifold structure for spiking-like regimes, generalize to a fundamentally different neuron type.

**Fig 5:**
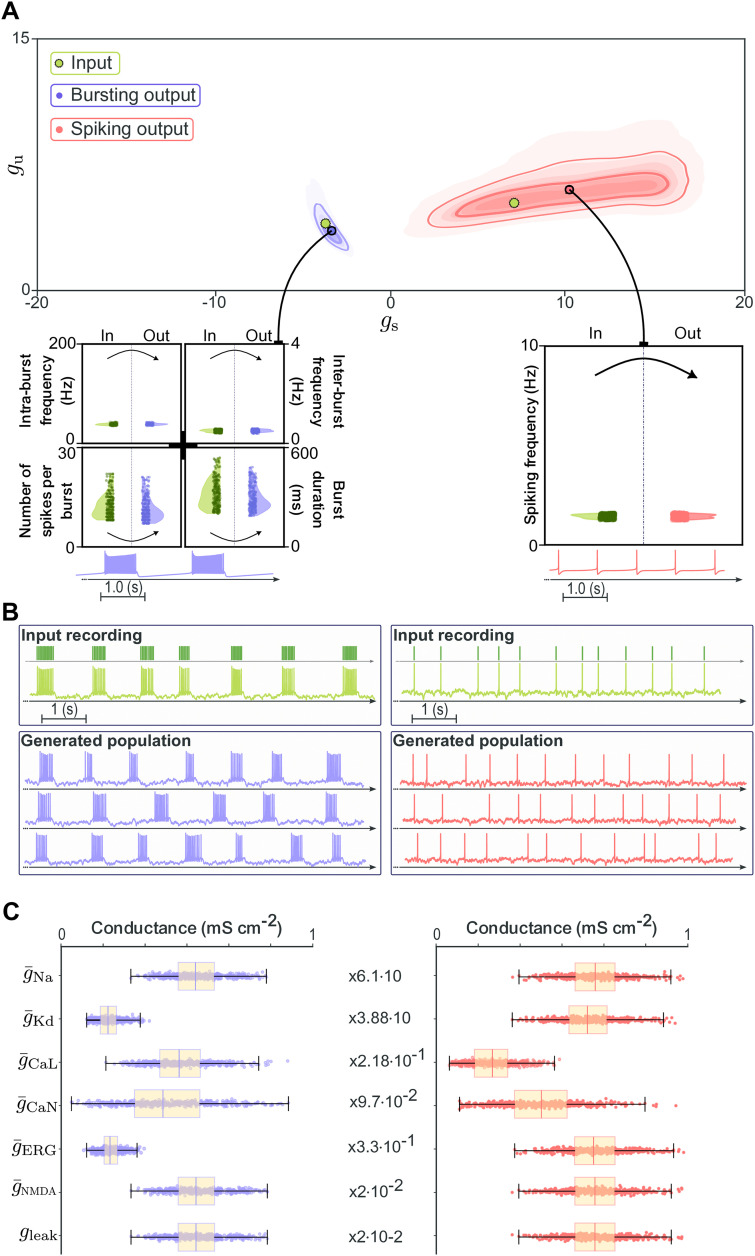
Performance of the adapted pipeline on the DA neuron model. **(A)** Two target DIC values (green dots) are selected in the DA $\left(g_{\text{s}},g_{\text{u}}\right)$ plane, one for each tested activity regime: slow pacemaking and bursting. For each target, the pipeline infers a conditional density over DICs (contour plots), from which DIC targets are sampled to generate output populations. Violin plots compare input and output activity descriptors across regimes. **(B)** Target spike trains (dark green, top) compared with three representative generated spike trains for slow pacemaking (right) and bursting (left), showing accurate reproduction of activity patterns. **(C)** Distributions of maximal conductances across generated neurons for both regimes, demonstrating broad parameter variability despite similar dynamics.

We evaluated the adapted pipeline using the same end-to-end protocol as for the STG model (Fig 5A). Two target DIC values were selected in the DA $\left(g_{\text{s}},g_{\text{u}}\right)$ plane, one per tested activity regime. For each target, an input population of 256 neurons was generated and passed through the full pipeline to produce an output population of equal size. Across both regimes, the inferred DIC densities are well localized and the output populations closely reproduce the firing statistics of the inputs, confirming that the pipeline maintains quantitative accuracy after transfer.

Qualitative inspection further confirms that the pipeline produces spike trains consistent with the target DA activity patterns across both tested regimes (Fig 5B). Representative input and generated traces show close temporal agreement for slow pacemaking and bursting. As in the STG case, the generated populations preserve degeneracy: despite producing similar output activity, the distributions of maximal conductances remain broad and heterogeneous (Fig 5C), reflecting the intrinsic degeneracy of the DA conductance space.

In summary, LoRA provides an efficient strategy for extending the pipeline to new CBMs with minimal retraining. Even across neuron models with fundamentally different conductance compositions and activity repertoires, the pipeline preserves both accuracy and degeneracy, highlighting the robustness of the DIC framework as an intermediate representation for generalization across models.

## Discussion

Our results demonstrate that combining deep learning with DIC theory enables the efficient and accurate reconstruction of degenerate conductance-based populations from spike times alone. The pipeline generalizes across activity regimes, stochastic inputs mimicking physiological conditions, and distinct conductance-based models, while consistently preserving both accuracy and degeneracy. In the following, we discuss the generality of the DIC framework, its relationship to existing inference approaches, its ability to capture degeneracy, current limitations, and potential experimental applications.

### DICs as a model-independent intermediate representation

A key feature of our approach is that DICs provide a model-independent intermediate representation. The DIC decomposition depends only on the structure of the dynamical system, not on its particular realization, and can therefore be computed for any model admitting a separation into fast, slow, and ultra-slow feedback processes. This includes detailed conductance-based models, reduced neuronal models (e.g., Morris-Lecar or FitzHugh-Nagumo type), and analog electronic circuits implementing neuromorphic dynamics [32,42]. Crucially, the first pipeline step (mapping spike times to DIC values) is entirely model-agnostic, as it operates solely on spike timing. Only the second step (generating conductance configurations from DIC constraints) requires a model-specific sensitivity matrix and compensation procedure. Extending the pipeline to a new model class therefore amounts to deriving the corresponding sensitivity matrix and implementing the appropriate compensation, while reusing the trained encoder or adapting it via transfer learning, as demonstrated with the LoRA-based adaptation from the STG to the DA model. This modularity positions the pipeline not as a tool tied to a specific model, but as a general framework applicable to any system in which neuronal excitability can be decomposed by timescale.

### Relationship to existing inference approaches

Our approach differs from classical SBI methods [23,24] and data assimilation techniques [29,31] in that it decomposes the problem through an interpretable intermediate rather than learning posteriors directly in the high-dimensional conductance space. While classical SBI methods successfully approximate posterior distributions over conductances and thereby provide families of plausible models, they do not explicitly reveal *why* certain parameter combinations yield similar outputs. In contrast, the DIC representation provides a mechanistic explanation: degenerate conductance sets produce similar activity because they share similar timescale-specific feedback properties, which is precisely what DICs capture. This perspective resonates with the “sloppy model” framework from systems biology [43,44], which shows that many parameter combinations in complex models are poorly constrained by data. In that analogy, DICs identify the neuroscience-specific “stiff” directions in parameter space, namely the timescale-specific feedback components that most strongly determine firing behavior, while the remaining “sloppy” directions correspond to the degenerate conductance combinations that the compensation algorithm explores.

### Capturing degeneracy

A fundamental question raised by our approach is whether the generated populations capture the “right amount” of degeneracy. The observed spread of maximal conductances, often varying by several-fold across instances, is consistent with biological variability reported in experimental studies of identified neurons [7,45]. Beyond degeneracy in the conductance space, the pipeline captures degeneracy within the DIC space itself. In spiking regimes, where slow and ultra-slow components act as overlapping negative feedbacks, multiple DIC configurations produce functionally indistinguishable activity. The learned density naturally reflects this ambiguity by assigning probability mass across the corresponding one-dimensional manifold, rather than collapsing onto a single point estimate. A complete validation would require comparing inferred conductance distributions against experimental measurements from populations of neurons exhibiting similar activity, which remains an important future direction.

### Future directions and limitations

An important consideration concerns the activity regimes covered by the training dataset. The synthetic data were generated by sampling DIC values within the region of parameter space associated with spontaneous activity, where $g_{\text{f}}(V_{\text{th}})$ is negative and regenerative fast feedback is sufficient to sustain firing without external drive. This restriction reflects both a biological priority, as many neurons of interest, including the STG and DA models studied here, fire spontaneously under physiological conditions, and a practical constraint inherent to spike-based inference: in the absence of spikes, no information is available, so neurons silent at rest cannot be characterized from spike times alone. Within this regime, $g_{\text{f}}(V_{\text{th}})$ lies within a restricted range, which is why the inference targets the slow and ultra-slow components $\left(g_{\text{s}}(V_{\text{th}});\;g_{\text{u}}(V_{\text{th}})\right)$ that fully describe firing patterns under spontaneous conditions.

A natural extension concerns the excitable regime, in which a neuron rests below threshold and produces sparse, irregular spikes when stochastic input occasionally crosses threshold. This regime corresponds to a different region of DIC space, with $g_{\text{f}}(V_{\text{th}})>0$, that is not represented in the current training distribution. The DIC framework itself imposes no theoretical obstacle: spontaneous and excitable regimes are distinguished by the sign of $g_{\text{f}}(V_{\text{th}})$ rather than by any fundamental property of the decomposition. Extending the pipeline to the excitable regime would therefore amount to enlarging the training distribution to span $g_{\text{f}}(V_{\text{th}})>0$, adding $g_{\text{f}}(V_{\text{th}})$ as a third inference target alongside $g_{\text{s}}(V_{\text{th}})$ and $g_{\text{u}}(V_{\text{th}})$, and extending the compensation step to enforce all three DIC components. To illustrate that the architecture is not intrinsically tied to the spontaneous regime, we applied the trained pipeline to sparse spike trains generated by injecting a strong hyperpolarizing stochastic current into otherwise spontaneously active neurons, producing inputs in an excitable-like regime that the method has not encountered during training (see Supporting Information, S1 Appendix). For both spiking and bursting examples, the pipeline returns spontaneously active populations whose firing mode (spiking versus bursting) matches that of the input, even though the exact firing statistics are not reproduced, since the inferred neurons remain in the spontaneous region of DIC space. Combined with stimulus-driven recordings ($I_{\text{ext}}\neq 0$) discussed below, retraining over an extended DIC space would allow the method to additionally recover the precise statistics of excitable-regime activity and broaden the range of biological preparations to which the method can be applied.

The method also has limitations beyond the regime restriction. First, its reliance on spike times makes it blind to subthreshold dynamics such as after-depolarizations or detailed interspike interval shapes. However, the DIC framework can naturally accommodate constraints at additional voltage points [32], and building datasets that incorporate DIC values measured at multiple voltages would allow the method to infer richer neuronal dynamics. Second, the spiking regime remains inherently harder to resolve in DIC space than the bursting regime. Training under stochastic current injection exposes the network to realistic temporal variability, but does not resolve this fundamental ambiguity, since the manifold structure arises from the timescale redundancy itself rather than from insufficient input diversity. Incorporating responses to controlled stimuli ($I_{\text{ext}}\neq 0$) could further constrain inference in this regime by probing the neuron under different input conditions. Third, while we currently infer only maximal conductances, this choice aligns with experimental reality where effective channel density is the primary variable under neuromodulatory or pharmacological manipulation [46,47]; extending the framework to kinetic parameters or structural inference remains possible in principle. Finally, at the network level, incorporating synaptic conductances into the DIC framework would account for both intrinsic and synaptic contributions to activity [48], potentially enabling real-time closed-loop neuromodulation [37] or online tuning of neuromorphic devices [42].

### Experimental and biomedical applications

Beyond parameter inference, our pipeline offers practical tools for experimental neuroscience. Measuring all ionic conductances in a single neuron is often impractical, as typically only a subset can be probed, and the conventional approach of averaging conductance values across populations can produce fragile or unrepresentative models [36]. In contrast, our framework allows experimentalists to reconstruct plausible conductance distributions in real time from spike recordings alone. This opens concrete experimental avenues: in neuromodulation studies, comparing inferred population distributions before and after application of a neuromodulator could reveal how modulation reshapes the conductance landscape and steers neurons between activity regimes. In pharmacological settings, the pipeline could predict which ion channel targets are most affected by a given compound by tracking how inferred DIC values and conductance distributions shift under drug application. For clinical neuroscience, the ability to rapidly generate mechanistic models from extracellular recordings could support patient-specific computational modeling, for instance in the context of deep brain stimulation or epilepsy monitoring. To facilitate adoption in these diverse settings, we release the full framework as open-source software with a graphical interface [34].

## Conclusion

In summary, by using DICs as an interpretable low-dimensional intermediate, the pipeline achieves both robustness and generalization across regimes and models. The generality of the DIC framework ensures that the approach extends to any system where excitability can be decomposed by timescale, providing a foundation for experimental strategies that probe how neurons exploit degeneracy to maintain reliable function.

## Materials and methods

### Conductance-based models

We use conductance-based models (CBMs) to simulate neuronal dynamics. CBMs describe the membrane potential *V* as a function of ionic and leak currents across the membrane:


$$\begin{array}{c} C\frac{dV}{dt}+g_{\text{leak}}(V-E_{\text{leak}})=-\sum_{i\in ℐ}{\bar{g}}_{i}m_{i}^{p_{i}}(V,t)h_{i}^{q_{i}}(V,t)(V-E_{i})+I_{\text{ext}}. \end{array}$$ (2)

Here, *C* is the membrane capacitance, set to $1\;\mu F\;{cm}^{-12}$. Each ionic current i∈ℐ is characterized by its maximal conductance ${\bar{g}}_{i}$, gating variables *m*_*i*_ and *h*_*i*_ raised to integer powers *p*_*i*_ and *q*_*i*_, and reversal potential *E*_*i*_. The leak current $I_{\text{leak}}=g_{\text{leak}}(V-E_{\text{leak}})$ models passive ion flow. We denote $\bar{g}=[{\bar{g}}_{1},\ldots ,{\bar{g}}_{\left|ℐ\right|},g_{\text{leak}}]\in {\mathbb{R}}_{+}^{N_{\text{model}}}=𝒢$ the vector of maximal conductances that distinguishes different instances of a model. The maximal conductances are the only parameters not treated as fixed constants in this work.

Gating variables $X\in \{m_{i},h_{i}\}$ follow first-order voltage-dependent dynamics:


$$\begin{array}{c} \tau_{X}(V)\frac{dX}{dt}=X_{\infty }(V)-X, \end{array}$$ (3)

where $\tau_{X}(V)$ is the voltage-dependent time constant and $X_{\infty }(V)$ is the steady-state value. To mimic physiological stochasticity, the external current *I*_ext_ is set to a low-pass filtered Gaussian noise signal with standard deviation $\sigma_{\text{noise}}=5\;\mu A\;{cm}^{-2}$ and cutoff frequency 1000 Hz.

As a proof of concept, we use two established neuron models. The stomatogastric ganglion (STG) neuron model [9] includes 7 ionic conductances (fast sodium, delayed rectifier potassium, calcium-dependent potassium, transient A-type potassium, slow calcium, transient T-type calcium, and hyperpolarization-activated cation) and explicitly incorporates intracellular calcium dynamics. The dopaminergic (DA) neuron model [49] includes 6 ionic conductances (fast sodium, delayed rectifier potassium, L-type calcium, N-type calcium, ERG potassium, and NMDA receptor-mediated). The STG model exhibits spiking and bursting, while the DA model additionally displays slow pacemaking and fast spiking. Detailed specifications and parameters for both models are provided in the Supporting Information (see S1 Appendix).

### Dynamic input conductances (DICs)

DICs, introduced in [32], provide a mathematically grounded framework for analyzing CBMs by decomposing the total membrane response into timescale-specific components.

### Timescale decomposition of membrane dynamics

The influence of membrane currents is partitioned into three characteristic temporal components. The fast dynamic conductance *g*_f_(*V*) governs the rapid voltage changes underlying spike upstroke. The slow conductance *g*_s_(*V*) regulates membrane repolarization and interspike interval behavior. The ultra-slow component *g*_u_(*V*) accounts for adaptation and shapes the bursting envelope. Each component can be computed analytically from the CBM equations (see Supporting Information, S1 Appendix), and the three are conveniently gathered in a sensitivity matrix *S*(*V*):


$$\begin{array}{c} g_{\text{DICs}}(V)=[\begin{array}{c} g_{\text{f}}(V)\\ g_{\text{s}}(V)\\ g_{\text{u}}(V) \end{array}]=S(V;\bar{g})\cdot \bar{g}. \end{array}$$ (4)

The sensitivity matrix summarizes the normalized influence of each conductance on the different timescales, scaled by the maximal conductances to obtain the dynamic conductances of a given instance. When *S* is independent of $\bar{g}$, the compensatory structure is *linear*; otherwise, it is *nonlinear*.

### Dimensionality reduction via DICs

In the DIC framework, neuronal excitability is largely characterized by the DIC values at a critical voltage called the threshold potential *V*_th_. Following [33], we approximate *V*_th_ as the first decreasing zero of the total conductance curve $g_{\text{t}}(V)=g_{\text{f}}(V)+g_{\text{s}}(V)+g_{\text{u}}(V)$. This zero-crossing has a dynamical interpretation: it marks the voltage at which the net feedback from all ionic conductances changes sign, transitioning from stable (restoring) to unstable (regenerative) dynamics. In many neuron models, this is closely related to the voltage at which a saddle-node bifurcation occurs in response to sustained input [50], making the neuron maximally sensitive to parameter changes and DIC values evaluated at *V*_th_ particularly informative about firing behavior. If a neuron model lacks sufficient regenerative conductances, the total conductance curve may not cross zero. In those cases, we use the mean threshold voltage across the population as a stable approximation (see Supporting Information, S1 Appendix).

Evaluating the DICs at *V*_th_ yields a compact representation:


$$\begin{array}{c} g_{\text{DICs}}(V_{\text{th}})=[\begin{array}{c} g_{\text{f}}(V_{\text{th}})\\ g_{\text{s}}(V_{\text{th}})\\ g_{\text{u}}(V_{\text{th}}) \end{array}]\in {\mathbb{R}}^{3}, \end{array}$$ (5)

transforming the high-dimensional parameter space $\bar{g}\in 𝒢$ into a low-dimensional space. Importantly, this mapping is not injective: many distinct conductance sets can yield the same DIC vector and therefore similar activity, enabling controlled exploration of degeneracy.

### Generating degenerate populations from DICs

The method introduced in [33] generates maximal conductance vectors $\bar{g}$ satisfying prescribed target DIC values $g_{\text{DICs}}^{\mathrm{target}}(V_{\text{th}})$. The conductance vector is partitioned into two subsets: $\bar{g}=[{\bar{g}}_{\text{random}};{\bar{g}}_{\text{comp.}}]$. The random subset is sampled from distributions extending beyond the biological range [7,33], while the compensable subset is determined by solving:


$$\begin{array}{c} S_{\text{comp.}}(V_{\text{th}})\cdot {\bar{g}}_{\text{comp.}}=g_{\text{DICs}}^{\text{target}}(V_{\text{th}})-S_{\text{random}}(V_{\text{th}})\cdot {\bar{g}}_{\text{random}}\;, \end{array}$$ (6)

where the sensitivity matrix has been decomposed as $S=[S_{\text{random}};S_{\text{comp.}}]$ following the partition of $\bar{g}$. Because different draws of ${\bar{g}}_{\text{random}}$ yield different valid solutions for ${\bar{g}}_{\text{comp.}}$, the procedure naturally produces a degenerate population from a single set of DIC constraints.

This linear compensation is exact when *S* does not depend on ${\bar{g}}_{\text{comp.}}$. However, it becomes inaccurate for models with nonlinear compensatory structure. For example, the STG model includes intracellular calcium dynamics that depend on calcium conductances, making $S=S(V;\bar{g})$. In [33], this was addressed by approximating *S* using fixed default values, which can introduce residual errors between target and enforced DIC values. To address this, we solve the compensation iteratively: the sensitivity matrix is recomputed at each step based on the current conductance estimates, and the linear system is resolved until convergence. In practice, five iterations reduce residuals by approximately a factor of 15 compared to the single-step method, with no convergence failures observed under physiological parameter ranges. Full details of the iterative procedure, convergence analysis, and the specific conductance partitions used for each model are provided in Supporting Information (see S1 Appendix).

### Synthetic dataset generation

**Fig 6:**
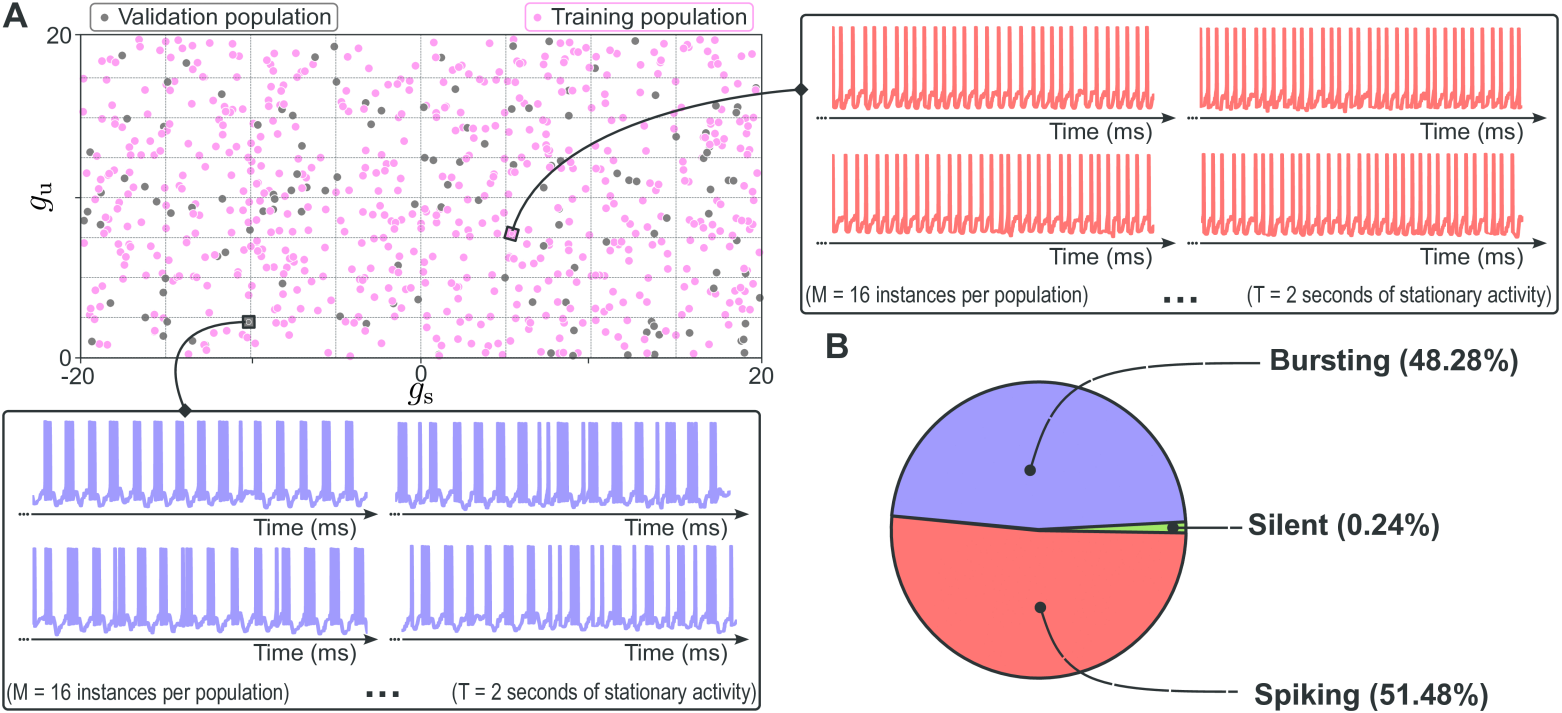
The synthetic dataset generation process from sampling in the DICs space. **(A)** Subset of the sampled DIC space used to generate degenerate CBM populations. Each dot corresponds to one population (16 instances), belonging to the training (pink) or validation (gray) set. Sampling is uniform and bounded to ensure broad coverage. Each population is simulated under noisy current injection, spike times are extracted, and descriptors are computed. Each instance is then classified as spiking, bursting, or silent. **(B)** Class distribution across the full dataset: 51.48% spiking, 48.28% bursting, and 0.24% silent.

We constructed a large open-source synthetic dataset spanning a broad range of DIC values and corresponding spike trains [39]. Instead of sampling conductance parameters directly, we uniformly sampled the slow and ultra-slow DICs $g^{*}=\left(g_{\text{s}}(V_{\text{th}});\;g_{\text{u}}(V_{\text{th}})\right)$ across bounded regions derived from extensive model exploration (Fig 6A), since these components primarily shape firing activity. For each of the *N* = 75,000 sampled DIC pairs, we generated a degenerate population of *M* = 16 CBM instances using the iterative compensation algorithm, yielding a total of |𝒯|=1,200,000 simulated neurons. Each instance was simulated under noisy current injection, and only spike times were retained from the resulting voltage traces:


$$\begin{array}{c} V(t)\overset{\text{transformed into}\;}{\underset{\;\;}{\to }}\;x=[t_{1},t_{2},\ldots ,t_{N_{\text{spikes}}}],\;t_{1}<t_{2}<\ldots <t_{N_{\text{spikes}}}. \end{array}$$

The final dataset consisted of 51.48% spiking and 48.28% bursting neurons, with 0.24% silent instances discarded (Fig 6B). For the DA model, a reduced dataset of approximately 40% of the STG size was used, since LoRA-based transfer learning facilitates adaptation with smaller datasets. Full details of the dataset construction, including sampling bounds, simulation parameters, activity classification criteria, and train/validation/test partitioning, are provided in Supporting Information (see S1 Appendix).

### Deep learning architecture and training

#### Problem formulation as amortized posterior approximation.

We frame the prediction of DIC values from spike trains as an amortized posterior approximation problem [24]. Rather than learning a point estimate, we aim to learn a parametric conditional density $p_{\theta }(g^{*}|x)$ over the slow and ultra-slow DIC targets $g^{*}=(g_{\text{s}}(V_{\text{th}});\;g_{\text{u}}(V_{\text{th}}))$, given a variable-length spike train $x=[t_{1},t_{2},\ldots ,t_{N_{\text{spikes}}}]$. This formulation naturally accounts for degeneracy in the DIC space, as multiple DIC configurations may be compatible with a given spike train. At inference time, plausible DIC targets are obtained by sampling from the learned conditional density. This poses two main challenges: (1) the input sequences are of variable length, while deep neural networks typically require fixed-size inputs; and (2) learning meaningful latent representations from raw spike data, without relying on manually engineered summary statistics, is a key design objective.

To address these challenges, we design an architecture combining an attention-based encoder that maps raw spike trains into a fixed-dimensional latent representation, and a normalizing flow decoder that parameterizes the conditional density over DIC values given this representation.

### Architecture overview

The architecture comprises an attention-based encoder [51] and a multi-headed decoder (see Supporting Information, S1 Appendix, for a detailed schematic and full specification).

The encoder first extracts inter-spike intervals (ISIs) and second-order ISI differences from the raw spike train, applies a logarithmic transform for numerical stability, and standardizes the resulting features. These are projected into a higher-dimensional embedding space with sinusoidal positional encoding, then processed by a stack of transformer blocks using multi-head self-attention. A self-attention pooling layer aggregates the variable-length representation into a fixed-size latent vector $z_{\text{latent}}\in {\mathbb{R}}^{d_{\text{latent}}}$.

The decoder operates on *z*_latent_ through three parallel heads. The primary head is a RealNVP-style normalizing flow [52] that models the conditional density $p_{\theta }(g^{*}|z_{\text{latent}})$, from which DIC targets are sampled at inference time. Two auxiliary heads, used only during training to regularize the encoder, perform classification of the firing regime (spiking or bursting) and regression of five activity descriptors (mean firing rate for spiking; intra-burst frequency, inter-burst frequency, burst duration, and spikes per burst for bursting). The auxiliary latent outputs are integrated with the encoder output via learnable element-wise mixing before being passed to the normalizing flow. At test time, only the normalizing flow head is used.

### Training procedure

The model is trained end-to-end by minimizing a composite loss combining the negative log-likelihood of the normalizing flow with weighted auxiliary losses: a masked mean squared error for the activity metrics regression and a balanced cross-entropy for firing regime classification.

To improve generalization, we apply three forms of data augmentation during training: (1) random cropping of spike trains to a window $D\sim 𝒰[N_{\text{spikes}}/2,N_{\text{spikes}}]$, (2) Gaussian noise $\epsilon_{i}\sim 𝒩(0,(2\;\text{ms}{)}^{2})$ added to spike times, and (3) 5% spike dropout. These augmentations simulate experimental variability and are applied independently for each training sample at each update.

Hyperparameters are optimized via random search over 100 configurations [53], using a lightweight pointwise regression decoder in place of the normalizing flow to reduce computational cost. The selected hyperparameters are then used to train the full model with the normalizing flow head. Optimization uses AdamW [54] and a cosine annealing learning rate schedule with warm restarts. The final architecture comprises approximately 150,000 trainable parameters. Full loss formulations and hyperparameter details are provided in the Supporting Information (see S1 Appendix).

### Evaluation metrics

Model evaluation is carried out at two levels. During hyperparameter optimization, we use the MAE of the pointwise regression decoder on the validation set ${𝒯}_{\text{val}}$ as the primary metric. For test-time evaluation on ${𝒯}_{\text{test}}$, we report performance on the auxiliary tasks: the regression head is evaluated using MAE on the activity descriptors, and the classification head using balanced accuracy. These auxiliary metrics verify that the encoder learns a meaningful and structured latent representation of the input spike trains.

The quality of the predicted DIC targets is ultimately assessed through a posterior predictive check (PPC) [55]: spike trains from held-out populations are passed through the full pipeline, and the resulting generated populations are compared against the inputs using key activity descriptors. This end-to-end evaluation directly measures whether the learned conditional density produces DIC samples that, once processed by the compensation algorithm, reproduce the observed firing patterns (Fig 4 and Fig 5). We additionally assess posterior calibration in the Supporting Information (see S1 Appendix) using TARP [56], simulation-based calibration rank histograms [57], and expected coverage tests [58].

### Transfer to the DA model

To adapt the pipeline to the DA neuron model, we apply parameter-efficient fine-tuning using Low-Rank Adaptation (LoRA) [41]. LoRA adapters are introduced in the linear layers of the network while attention layers remain unmodified. The majority of parameters from the STG-trained backbone are frozen, and only the newly introduced LoRA parameters are updated; the input normalization layer is recalculated based on the DA training set. The DA dataset is generated using the same procedure as for the STG model, yielding approximately 25,000 active populations after discarding silent instances. Full details are provided in Supporting Information (see S1 Appendix).

## Supporting information

S1 Appendix Details and additional experiments. We provide all elements required to reproduce the results presented in this paper, as well as additional experiments. This includes model equations, DIC derivations, compensation procedure details, sampling ranges, hyperparameter tuning, architecture schematics, convergence analysis, and posterior calibration diagnostics.
(PDF)
