# Supplementary material for: Fast reconstruction of degenerate populations of conductance-based neuron models from spike times
Source: PLoS Comput Biol. 2026 May 21;22(5):e1014337. doi: 10.1371/journal.pcbi.1014337 (PMC13241015; doi:10.1371/journal.pcbi.1014337)
Supplement: S1 Appendix — We provide all elements required to reproduce the results presented in this paper, as well as additional experiments. This includes model equations, DIC derivations, compensation procedure details, sampling ranges, hyperparameter tuning, architecture schematics, convergence analysis, and posterior calibration diagnostics. (PDF) [file pcbi.1014337.s001.pdf]

S1 Appendix

Fast reconstruction of degenerate populations of conductance-based neuron models from spike times

We present all the elements needed in addition to the main text to reproduce the results reported in this paper. We also provide additional experiments that complement the main findings.

A Conductance-based models

This section details the equations of the conductance-based models (CBMs) used in this work. The stomatogastric ganglion (STG) model was adapted from [S1], and the dopaminergic (DA) model was adapted from [S2].

Both models follow a common structure in which the membrane potential V evolves according to an ordinary differential equation involving ionic and leak currents:

$$C\frac{dV}{dt} + g_{\text{leak}}(V - E_{\text{leak}}) = -\sum_{i\in\mathcal{I}} \bar{g}_i m_i^{p_i}(V,t) h_i^{q_i}(V,t)(V - E_i) + I_{\text{ext}} \quad . \tag{S1}$$

Here, C is the membrane capacitance, set to $1\,\mu\text{F}\,\text{cm}^{-2}$ in both models. The set $\mathcal{I}$ contains all ionic conductances considered for a given model, detailed below for each case. Each ionic current is characterized by its maximal conductance $\bar{g}_i$ and by gating variables m_i (activation) and h_i (inactivation), each raised to integer powers p_i and q_i, respectively. E_i is the Nernst reversal potential for the associated ion. The leak current is modeled as $I_{\text{leak}} = g_{\text{leak}}(V - E_{\text{leak}})$. The external input current I_{ext} is set to a low-pass filtered Gaussian noise signal with standard deviation $\sigma_{\text{noise}} = 5\,\mu\text{A}\,\text{cm}^{-2}$ and cutoff frequency 1000 Hz, as described in the main text. We denote $\bar{g} = [\bar{g}_1,\ \bar{g}_2,\ \ldots,\ \bar{g}_{|\mathcal{I}|}, g_{\text{leak}}] \in \mathbb{R}^{N_{\text{model}}}$ the vector of maximal conductances (extended by the leak conductance) that defines different instances of a model. The maximal conductances are the only parameters varied in this work.

Gating variables $X \in \{m_i, h_i\}$ are dimensionless quantities constrained between 0 and 1, representing the fraction of ion channels that are activated (m_i) or not inactivated (h_i). They follow first-order voltage-dependent dynamics of the form:

$$\tau_X(V)\frac{dX}{dt} = X_\infty(V) - X \quad , \tag{S2}$$

where $\tau_X(V)$ is the voltage-dependent time constant and $X_\infty(V)$ is the steady-state activation or inactivation value.

Simulations are implemented in Python using the SciPy library [S3], solving the system of differential equations with the BDF solver [S4], well suited for stiff systems with multiple timescales.

The maximum time step is set to 0.05 ms. Simulations are parallelized across CPU cores to accelerate dataset generation.

A.1 The stomatogastric ganglion neuron model

The STG model [S1] includes eight ionic currents: the fast transient sodium current (Na), the delayed rectifier potassium current (Kd), the calcium-activated potassium current (KCa), the A-type potassium current (A), the slow calcium current (CaS), the transient calcium current (CaT), the hyperpolarization-activated current (H), and the leak current. In addition to the standard voltage-dependent gating, this model explicitly incorporates intracellular calcium concentration dynamics through an additional ordinary differential equation:

$$\tau_{\mathrm{Ca}} \frac{d\mathrm{Ca}}{dt} = -\alpha_{\mathrm{Ca}} \left(I_{\mathrm{CaS}} + I_{\mathrm{CaT}} \right) - \mathrm{Ca} + \beta_{\mathrm{Ca}} \quad ,$$

which modulates calcium-dependent potassium currents through a voltage- and calcium-dependent steady-state gating function $m_{\infty,\mathrm{KCa}}(V, \mathrm{Ca})$. This coupling introduces a nonlinear dependence between conductance parameters and the sensitivity matrix, motivating the iterative compensation algorithm described in Section C.

Table S1 provides the values of the fixed model parameters. As initial conditions, we used $V_0 = -70$ mV and $\mathrm{Ca}_0 = 0.5$ µM, with gating variables initialized at steady state: $X_0 = X_\infty(V_0; \mathrm{Ca}_0)$. The first 3000 ms of each simulation were discarded to avoid transient effects. Table S3 lists the mathematical expressions for the steady-state gating functions, the time constant functions, and the corresponding gating exponents.

Table S1. Fixed parameters for the STG model. Reversal potentials, calcium time constant, and calcium dynamics parameters used in the STG model.

E_{leak}	E_{Na}	E_{K}	E_{H}	E_{Ca}	τ_{Ca}	α_{Ca}	β_{Ca}
−50 mV	50 mV	−80 mV	−20 mV	80 mV	20 ms	0.94 mM nF nA^{-1}	0.05 µM

A.2 The dopaminergic neuron model

The DA model [S2] includes seven ionic currents: the fast sodium current (Na), the delayed rectifier potassium current (Kd), the ERG potassium current (ERG), the L-type calcium current (CaL), the N-type calcium current (CaN), the NMDA receptor-mediated current (NMDA), and the leak current. This model incorporates a magnesium-sensitive current through the NMDA channel, influenced by a fixed extracellular magnesium concentration.

The NMDA current is treated as instantaneous and always evaluated at its steady-state value:

$$I_{\mathrm{NMDA}} = \bar{g}_{\mathrm{NMDA}}(V - E_{\mathrm{NMDA}}) \cdot m_{\mathrm{NMDA},\infty}(V, \mathrm{Mg}) \quad ,$$

where $m_{\mathrm{NMDA},\infty}$ is the voltage- and magnesium-dependent steady-state activation function.

For the ERG channel, the gating variables o_{ERG} and i_{ERG} evolve according to the differential equations:

$$\frac{do_{\mathrm{ERG}}}{dt} = a_0(V) \cdot (1 - o_{\mathrm{ERG}} - i_{\mathrm{ERG}}) + b_i(V) \cdot i_{\mathrm{ERG}} - o_{\mathrm{ERG}} \cdot (a_i(V) + b_0(V)) \quad ,$$

$$\frac{di_{\mathrm{ERG}}}{dt} = a_i(V) \cdot o_{\mathrm{ERG}} - b_i(V) \cdot i_{\mathrm{ERG}} \quad .$$

The steady-state expressions for the ERG gating variables under constant voltage V are:

$$o_{\mathrm{ERG},\infty}(V) = \frac{a_0(V) \cdot b_i(V)}{a_0(V) \cdot (a_i(V) + b_i(V)) + b_0(V) \cdot b_i(V)} \quad ,$$

$$i_{\mathrm{ERG},\infty}(V) = \frac{a_0(V) \cdot a_i(V)}{a_0(V)(a_i(V) + b_i(V)) + b_0(V) \cdot b_i(V)} \quad .$$

The corresponding ERG current is:

$$I_{\mathrm{ERG}} = \bar{g}_{\mathrm{ERG}} \cdot o_{\mathrm{ERG}} \cdot (V - E_K) \quad .$$

Table S2 provides the fixed model parameters. As initial conditions, we used $V_0 = -90\,\mathrm{mV}$, with gating variables initialized at steady state: $X_0 = X_\infty(V_0)$. The first 3000 ms of each simulation were discarded to avoid transient effects. Table S4 lists the mathematical expressions for all gating functions and kinetic parameters.

Table S2. Fixed parameters for the DA model. Reversal potentials and magnesium concentration used in the DA model.

E_{Na}	E_{K}	E_{Ca}	E_{leak}	E_{NMDA}	Mg
60 mV	−85 mV	60 mV	−50 mV	0 mV	1.4

Table S3. Gating functions and kinetics for the STG model. Steady-state activation/inactivation functions, time constants, and exponents for each ionic current in the STG model. All f functions refer to the generalized sigmoid function (Eq. S3).

Current I_i	p_i	q_i	$m_{i,\infty}(V)$ or $m_{i,\infty}(V,\mathrm{Ca})$	$h_{i,\infty}(V)$	$\tau_{m_i}(V)$	$\tau_{h_i}(V)$
I_{Na}	3	1	$f(V,0,1,-5.29,25.5)$	$f(V,0,1,5.18,48.9)$	$f(V,1.32,-1.26,-25,120)$	$f(V,0,0.67,-10,62.9) \cdot f(V,1.5,1,3.6,34.9)$
I_{Kd}	4	0	$f(V,0,1,-11.8,12.3)$	—	$f(V,7.2,-6.4,-19.2,28.3)$	—
I_{CaT}	3	1	$f(V,0,1,-7.2,27.1)$	$f(V,0,1,5.5,32.1)$	$f(V,21.7,-21.3,-20.5,68.1)$	$f(V,105,-89.8,-16.9,55)$
I_{CaS}	3	1	$f(V,0,1,-8.1,33)$	$f(V,0,1,6.2,60)$	$1.4+\frac{7}{\exp\left(\frac{V+27}{10}\right)+\exp\left(\frac{V+70}{-13}\right)}$	$60+\frac{150}{\exp\left(\frac{V+55}{9}\right)+\exp\left(\frac{V+65}{-16}\right)}$
I_{KCa}	4	0	$\frac{\mathrm{Ca}}{\mathrm{Ca}+3} \cdot f(V,0,1,-12.6,28.3)$	—	$f(V,90.3,-75.1,-22.7,46)$	—
I_{A}	3	1	$f(V,0,1,-8.7,27.2)$	$f(V,0,1,4.9,56.9)$	$f(V,11.6,-10.4,-15.2,32.9)$	$f(V,38.6,-29.2,-26.5,38.9)$
I_{H}	1	0	$f(V,0,1,6,70)$	—	$f(V,272,1499,-8.73,42.2)$	—
I_{leak}	0	0	—	—	—	—

Table S4. Gating functions and kinetics for the DA model. All f functions refer to the generalized sigmoid function (Eq S3). Additionally, there is a current I_{ERG} described by the ERG channel, whose rate functions are defined as exponentials of the membrane potential V. The activation and inactivation parameters are as follows: $a_0(V) = 0.0036\exp(0.0759V)$, $b_0(V) = 1.2523 \times 10^{-5}\exp(-0.0671V)$, $a_i(V) = 0.1\exp(0.1189V)$, and $b_i(V) = 0.003\exp(-0.0733V)$.

Current	p_i	q_i	$m_{i,\infty}(V)$ or $m_{i,\infty}(V,\mathrm{Mg})$	$h_{i,\infty}(V)$	$\tau_{m_i}(V)$	$\tau_{h_i}(V)$
I_{Na}	3	1	$f(V,0,1,-9.7264,30.0907)$	$f(V,0,1,10.7665,54.0289)$	$0.01+\frac{1.0}{(-\frac{15.6504+0.4043V}{\exp(-19.565-0.5052V)-1.0})+3.0212\exp(-0.007463V)}$	$0.4+\frac{1.0}{(0.00050754\exp(-0.063213V))+9.7529\exp(0.13442V)}$
I_{Kd}	3	0	$f(V,0,1,-12,25)$	—	$f(V,20,-18,-10,38)$	—
I_{CaL}	2	0	$f(V,0,1,-2,50)$	—	$f(V,30,-28,-3,45)$	—
I_{CaN}	1	0	$f(V,0,1,-7,30)$	—	$f(V,30,-25,-6,55)$	—
I_{NMDA}	1	0	$\frac{1}{1+\frac{\mathrm{Mg}\cdot\exp(-0.08V)}{10}}$	—	—	—
I_{leak}	0	0	—	—	—	—

$$f(V,A,B,C,D) = A + \frac{B}{1+\exp\left(\frac{V+D}{C}\right)} \tag{S3}$$

B Dynamic input conductances (DICs)

This work uses the concept of Dynamic Input Conductances (DICs) [S5], which consist of voltage-dependent conductances separated according to timescales. We use three timescales: fast, slow, and ultra-slow. These three DIC components have been shown to be sufficient to qualitatively determine excitability in neuron models [S5, 6]. Specifically, based on DIC values at threshold, it becomes possible to predict the firing pattern of a neuron. The computation follows recent work on the subject [S6, 7].

We denote $g_{\mathrm{f}}(V)$, $g_{\mathrm{s}}(V)$, and $g_{\mathrm{u}}(V)$ the fast, slow, and ultra-slow DIC components, respectively. The total DIC is $g_{\mathrm{t}}(V) = g_{\mathrm{f}}(V) + g_{\mathrm{s}}(V) + g_{\mathrm{u}}(V)$. In this work, we focus on a particular voltage value, the threshold voltage V_{th}. As an approximation, we consider V_{th} to be a fixed value shared by all instances of a given CBM. We make this approximation mainly because computing V_{th} requires knowing $\bar{g}$, which is unknown in advance. The choice of V_{th} is discussed in Section C.3.

B.1 Computation details

In CBMs, the timescale-specific conductances can be computed analytically:

$$\begin{cases} g_{\mathrm{f}}(V) &= \left[-\dfrac{\partial \dot{V}}{\partial V} - \sum_i w_{\mathrm{fs},X_i}(V) \left(\dfrac{\partial \dot{V}}{\partial X_i} \dfrac{\partial X_{i,\infty}}{\partial V} \right) \right] \Bigg|_V \dfrac{1}{g_{\mathrm{leak}}} \quad , \\ g_{\mathrm{s}}(V) &= \left[- \sum_i \left(w_{\mathrm{su},X_i}(V) - w_{\mathrm{fs},X_i}(V) \right) \left(\dfrac{\partial \dot{V}}{\partial X_i} \dfrac{\partial X_{i,\infty}}{\partial V} \right) \right] \Bigg|_V \dfrac{1}{g_{\mathrm{leak}}} \quad , \\ g_{\mathrm{u}}(V) &= \left[- \sum_i \left(1 - w_{\mathrm{su},X_i}(V) \right) \left(\dfrac{\partial \dot{V}}{\partial X_i} \dfrac{\partial X_{i,\infty}}{\partial V} \right) \right] \Bigg|_V \dfrac{1}{g_{\mathrm{leak}}} \quad , \end{cases} \tag{S4}$$

where the terms X_i correspond to gating variables (m_i or h_i) controlling activation and inactivation of ion channels, and $w_{\mathrm{fs},X_i}(V)$ and $w_{\mathrm{su},X_i}(V)$ are voltage-dependent weighting factors. The voltage dependence is omitted in the right-hand sides for readability. The sign convention and normalization by the leak conductance follow [S6], and thus differ from the original formulation in [S5].

Weighting functions

The weighting functions determine how each gating variable contributes to different timescales, based on a logarithmic scaling between 0 and 1 using reference timescales:

$$w_{\mathrm{fs},X_i}(V) = \begin{cases} 1 & , \quad \tau_{X_i}(V) \leq \tau_{\mathrm{f}}(V) \quad , \\ \dfrac{\log(\tau_{\mathrm{s}}(V)) - \log(\tau_{X_i}(V))}{\log(\tau_{\mathrm{s}}(V)) - \log(\tau_{\mathrm{f}}(V))} & , \quad \tau_{\mathrm{f}}(V) < \tau_{X_i}(V) \leq \tau_{\mathrm{s}}(V) \quad , \\ 0 & , \quad \tau_{X_i}(V) > \tau_{\mathrm{s}}(V) \quad , \end{cases}$$

$$w_{\mathrm{su},X_i}(V) = \begin{cases} 1 & , \quad \tau_{X_i}(V) \leq \tau_{\mathrm{s}}(V) \quad , \\ \dfrac{\log(\tau_{\mathrm{u}}(V)) - \log(\tau_{X_i}(V))}{\log(\tau_{\mathrm{u}}(V)) - \log(\tau_{\mathrm{s}}(V))} & , \quad \tau_{\mathrm{s}}(V) < \tau_{X_i}(V) \leq \tau_{\mathrm{u}}(V) \quad , \\ 0 & , \quad \tau_{X_i}(V) > \tau_{\mathrm{u}}(V) \quad . \end{cases} \tag{S5}$$

The reference timescales τ_{f}, τ_{s}, and τ_{u} are chosen according to the characteristic timescales of bursting and spiking dynamics [S5]. Specifically, τ_{f} corresponds to the activation time constant of the fastest depolarizing current, τ_{s} represents that of the fastest repolarizing current, and τ_{u} is associated with the slowest variable in the system, typically governing burst adaptation.

For the STG model, we used $\tau_{m_{\mathrm{Na}}}(V)$, $\tau_{m_{\mathrm{Kd}}}(V)$, and $\tau_{\mathrm{H}}(V)$, respectively. For the DA model, we used $\tau_{m_{\mathrm{Na}}}(V)$, $\tau_{m_{\mathrm{Kd}}}(V)$, and a constant function $\tau_{\mathrm{u,DA}} = 100\,\mathrm{ms}$.

Partial derivatives for standard channels

In the standard case of purely voltage-dependent ion channels, the terms $\frac{\partial \dot{V}}{\partial X_i}$ take the form:

$$\frac{\partial \dot{V}}{\partial m_i} = \bar{g}_i p_i m_{i,\infty}^{p_i-1} h_{i,\infty}^{q_i} \quad ; \qquad \frac{\partial \dot{V}}{\partial h_i} = \bar{g}_i q_i m_{i,\infty}^{p_i} h_{i,\infty}^{q_i-1} \quad . \tag{S6}$$

Sensitivity matrix formulation

A compact way of writing Eq. S4 is through the sensitivity matrix $S(V;\bar{g})$:

$$g_{\mathrm{DICs}}(V) = \begin{bmatrix} g_{\mathrm{f}}(V) \\ g_{\mathrm{s}}(V) \\ g_{\mathrm{u}}(V) \end{bmatrix} = S(V;\bar{g}) \cdot \bar{g} \quad . \tag{S7}$$

This is the formulation used in the generation procedure. One can construct the sensitivity matrix element by element by computing:

$$S_{j,i} = \frac{g_j^{(i)}}{\bar{g}_i}, \quad (i,j) \in \{1,2,\ldots,N_{\mathrm{model}}\} \times \{\mathrm{f},\mathrm{s},\mathrm{u}\} \quad , \tag{S8}$$

where $g_j^{(i)}$ corresponds to the partial sum from Eq. S4 involving only current i (that is, keeping only the terms containing $\bar{g}_i$, as in Eq. S6) for the DIC component associated with timescale j.

When S is independent of $\bar{g}$, the compensatory structure is *linear*; otherwise, it is *nonlinear*. In the STG model, intracellular calcium dynamics introduce a dependence of S on calcium conductances, resulting in nonlinear compensatory structure. The DA model has linear compensatory structure.

C Population generation procedure

In this work, we improved the procedure described in [S6] to generate degenerate populations of CBMs. The improved method, called the iterative compensation algorithm, is detailed below. The procedure is used both during dataset generation (Fig 6 from the main text) and at inference time once the deep learning architecture has been trained (Fig 3–6 from the main text). The core idea is to impose DIC values at the threshold voltage, as these are known to shape the firing pattern [S5, 6].

C.1 Compensation framework

The compensation procedure exploits the relationship $g_{\mathrm{DICs}}(V_{\mathrm{th}}) = S(V_{\mathrm{th}};\bar{g}) \cdot \bar{g}$ between DICs and maximal conductances. The conductance vector is partitioned into two subsets: $\bar{g} = [\bar{g}_{\mathrm{random}};\ \bar{g}_{\mathrm{comp.}}]$, where this partition preserves the total number of conductances, i.e., both subsets together contain all N_{model} components of $\bar{g}$. The random subset $\bar{g}_{\mathrm{random}}$ is sampled from distributions extending beyond the biological range [S6, 8], while the compensable subset $\bar{g}_{\mathrm{comp.}}$ is adjusted to satisfy the DIC constraints by solving:

$$S_{\mathrm{comp.}}(V_{\mathrm{th}}) \cdot \bar{g}_{\mathrm{comp.}} = g_{\mathrm{DICs}}^{\mathrm{target}}(V_{\mathrm{th}}) - S_{\mathrm{random}}(V_{\mathrm{th}}) \cdot \bar{g}_{\mathrm{random}} \quad , \tag{S9}$$

where the sensitivity matrix has been decomposed as $S = [S_{\mathrm{random}};\ S_{\mathrm{comp.}}]$ following the partition of $\bar{g}$. Because different draws of $\bar{g}_{\mathrm{random}}$ yield different valid solutions for $\bar{g}_{\mathrm{comp.}}$, the procedure naturally produces a degenerate population from a single set of DIC constraints.

This linear compensation is exact when S does not depend on $\bar{g}_{\mathrm{comp.}}$. However, it becomes inaccurate for models with nonlinear compensatory structure. In the STG model, intracellular

calcium dynamics depend on calcium conductances, making $S = S(V; \bar{g})$. In [S6], this was addressed by approximating S using fixed default values, which can introduce residual errors between target and enforced DIC values.

Iterative extension

To improve constraint satisfaction, we solve the compensation iteratively: starting from an initial guess $\bar{g}^{(0)}_{\text{comp.}}$, the sensitivity matrix is recomputed at each iteration based on the current conductance estimates, and a new linear system is solved:

$$A(\bar{g}^{(k)}_{\text{comp.}}) \cdot \bar{g}^{(k+1)}_{\text{comp.}} = b(\bar{g}^{(k)}_{\text{comp.}}), \quad k = 0, \ldots, K-1 \quad , \tag{S10}$$

where $A := S_{\text{comp.}}(V_{\text{th}})$ and $b := g^{\text{target}}_{\text{DICs}}(V_{\text{th}}) - S_{\text{random}}(V_{\text{th}}) \cdot \bar{g}_{\text{random}}$ are updated at each iteration. The process is repeated until the residual norm between target and actual DIC values becomes sufficiently small.

We chose this iterative fixed-point approach over general-purpose nonlinear optimization methods for several reasons. First, under physiological conditions, the procedure reliably converges within a small number of iterations ($K = 5$ in this work), making more sophisticated techniques unnecessary. Second, each iteration requires only solving a linear system, which is computationally inexpensive. Third, the method naturally preserves the structure of the original compensation framework, ensuring that the generated populations remain consistent with DIC theory. In extensive testing across both the STG and DA models, we observed no convergence failures under physiological parameter ranges.

Two-step compensation structure

The full generation procedure consists of two compensation steps performed in series. In the first step, $n = 3$ DIC constraints (fast, slow, and ultra-slow) are imposed to ensure spontaneous activity [S6]. For the STG model, this step adjusts one conductance contributing predominantly to each timescale: $\bar{g}_{\text{comp., spont.}} = (g_{\text{Na}}, g_{\text{Kd}}, g_{\text{H}})$, corresponding to fast, slow, and ultra-slow feedback respectively, while the remaining conductances are randomly sampled. For the DA model, the compensated set is $\bar{g}_{\text{comp., spont.}} = (g_{\text{Na}}, g_{\text{CaN}}, g_{\text{ERG}})$.

The second step refines the slow and ultra-slow DIC values toward the target activity ($n = 2$ constraints). Starting from the conductance vector obtained after the first step, a new partition $\bar{g} = [\bar{g}'_{\text{random}};\ \bar{g}'_{\text{comp.}}]$ is defined. Crucially, this partition can differ from the first step: conductances that were compensated initially may now be held fixed (becoming part of $\bar{g}'_{\text{random}}$), while different conductances are adjusted to satisfy the new constraints. This flexibility allows the method to target specific DIC components independently. In the STG model, the second step adjusts $\bar{g}'_{\text{comp.}} = (g_{\text{CaS}}, g_{\text{H}})$ when $g_{\text{s}} < 0$ (bursting regime) or $\bar{g}'_{\text{comp.}} = (g_{\text{A}}, g_{\text{H}})$ when $g_{\text{s}} > 0$ (spiking regime). In the DA model, the second step adjusts $\bar{g}'_{\text{comp.}} = (g_{\text{ERG}}, g_{\text{CaL}})$ when $g_{\text{s}} < 0$ or $\bar{g}'_{\text{comp.}} = (g_{\text{ERG}}, g_{\text{Kd}})$ when $g_{\text{s}} > 0$. Since this step starts from the output of the first compensation, no additional random sampling is performed.

C.2 Validation of the iterative compensation algorithm

To validate the iterative compensation algorithm, we compared its performance against the single-step linear compensation method from [S6]. We evaluated both methods on 1,633 target DIC configurations, generating $P = 250$ instances per target. For each target, we computed the mean residual norm across all generated instances, defined as the Euclidean distance between the target DICs and those actually enforced by the generated conductance vector:

$$\|r\|_2 = \left\| g^{\text{target}}_{\text{DICs}}(V_{\text{th}}) - S(V_{\text{th}}; \bar{g}) \cdot \bar{g} \right\|_2 \quad . \tag{S11}$$

Fig S1A compares the mean residual norms obtained with the linear method (hatched, corresponding to 0 iterations) and the iterative compensation algorithm (plain) over up to 10 iterations. After only five iterations, residuals were reduced by a factor of 15, striking a balance between accuracy and computational cost. Accordingly, we used five iterations as the default throughout the study.

Since DICs serve as an intermediate representation rather than the final target, a more direct measure of accuracy is given by the firing pattern distributions of the generated populations. Fig S1B–C compare populations generated with the linear (hatched) and iterative (plain) methods for spiking neurons (red, Fig S1B) and bursting neurons (purple, Fig S1C). The iterative algorithm reduces outliers and produces more compact, centered distributions of activity statistics. Spiking activity is summarized by mean firing frequency, while bursting activity is described by intra-burst frequency, inter-burst frequency, burst duration, and spikes per burst. These tighter distributions confirm that the iterative approach enhances the reliability of the DIC-based generative method.

Importantly, the iterative compensation algorithm preserves degeneracy. Sampling of $\bar{g}_{\mathrm{random}}$ introduces variability across instances, resulting in distinct compensation problems even for the same target DIC. This ensures that maximal conductance vectors remain heterogeneous, often differing by several folds, while still producing consistent activity statistics. This is illustrated in both spiking (Fig S1D) and bursting (Fig S1E) populations, where conductance distributions span wide ranges despite similar firing patterns.

These results demonstrate that the iterative compensation algorithm substantially improves constraint satisfaction while preserving the intrinsic biological heterogeneity characteristic of degenerate neuronal populations. The method forms the basis for generating the synthetic training dataset and is also employed during inference to map predicted DIC values to conductance-based model populations.

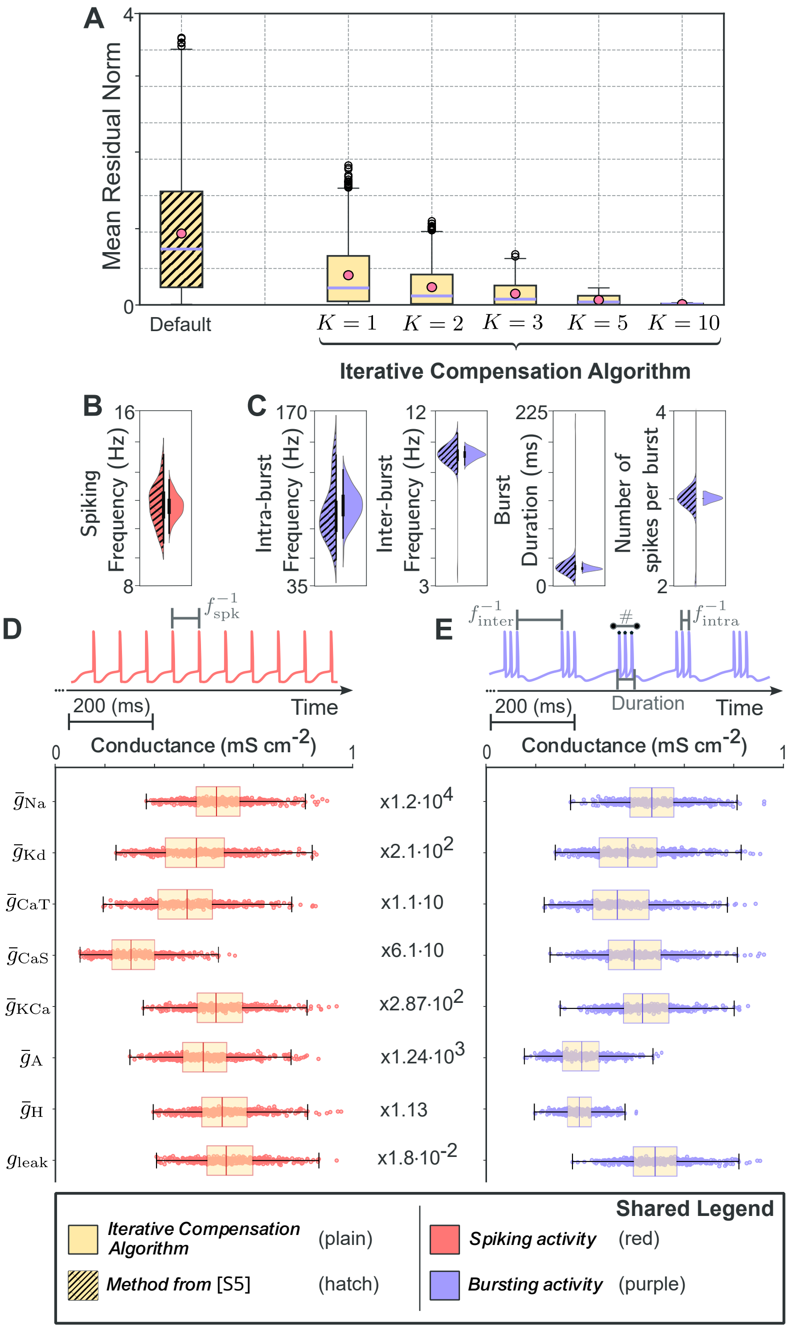

Fig S1. Iterative compensation improves constraint satisfaction and preserves degeneracy in CBMs with nonlinear dynamics. (A) Mean residuals of DIC constraints across 1,633 targets and 250 instances per population, comparing linear compensation (hatched, 0 iterations from [S6]) to iterative compensation (plain), showing a rapid reduction in residuals. **(B)** Distribution of mean firing frequency f_{spk} in spiking neurons (red), demonstrating tighter and more consistent activity with iterative compensation (plain) compared to linear (hatched). **(C)** Distributions of bursting features (intra-burst frequency f_{intra}, inter-burst frequency f_{inter}, burst duration, spikes per burst #) in bursting neurons (purple), showing more compact activity profiles with iterative compensation. **(D, E)** Maximal conductance values across spiking (D) and bursting (E) neuron populations generated with iterative compensation, revealing high variability despite similar firing patterns, illustrating preserved degeneracy.

C.3 Approximation of the threshold voltage

The generation procedure requires evaluating sensitivities at a fixed voltage V_{th}. However, the actual threshold voltage for a given conductance vector $\bar{g}$ is unknown before $\bar{g}$ is determined. We therefore approximate V_{th} by a constant value shared across all instances of a given CBM.

The threshold potential is defined as the first decreasing zero of the total conductance curve:

$$g_{\mathrm{t}}(V_{\mathrm{th}}) = 0 \quad \text{with} \quad g_{\mathrm{t}}(V_{\mathrm{th}} - \delta V) > 0 > g_{\mathrm{t}}(V_{\mathrm{th}} + \delta V), \quad \text{for all sufficiently small } \delta V > 0 \quad . \tag{S12}$$

To justify the shared-value approximation, we sampled 4,000 conductance vectors from $\mathcal{D}_{\mathrm{analysis}}$ (see Section C.4) and computed individual threshold voltages using Eq. S12 via a bisection method. The resulting distributions are narrow and unimodal, yielding $V_{\mathrm{th}} \approx -51\,\mathrm{mV}$ for the STG model and $V_{\mathrm{th}} \approx -55.5\,\mathrm{mV}$ for the DA model. These estimates are robust to sample size: repeating the analysis with 8,000 instances produced nearly identical values. Figure S2 shows the histogram obtained for 8,000 STG instances, confirming that most threshold values cluster tightly around the estimate.

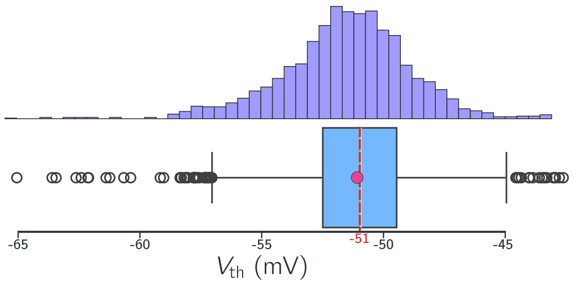

Fig S2. Histogram of threshold voltage estimates (V_{th}) for 8,000 STG model instances. The distribution remains nearly identical to that obtained with 4,000 instances, indicating convergence around $V_{\mathrm{th}} \approx -51\,\mathrm{mV}$. The mean (pink dot) and median (yellow line) are close to the estimated value (red line).

C.4 Conductance distributions

In practice, we use two distinct distributions of maximal conductances, denoted $\bar{g} \sim \mathcal{D}$, each serving a different purpose:

- $\mathcal{D}_{\mathrm{analysis}}$: a broad distribution covering a wide region of the conductance space, used for preliminary analyses (threshold voltage estimation, DIC space exploration) prior to population generation.

- $\mathcal{D}_{\mathrm{generation}}$: a more concentrated distribution, specifically employed during generation of degenerate populations.

The rationale for using two distributions stems from their different objectives. The distribution $\mathcal{D}_{\mathrm{analysis}}$ is designed to explore a wide range of instances and observe various properties of the CBM under study, requiring broad coverage to avoid losing information. The distribution $\mathcal{D}_{\mathrm{generation}}$ defines a parameter space that leads to degenerate behaviors: it should be spread enough to allow for significant degeneracy while minimizing the impact on firing variability. The variability in firing patterns when enforcing DIC values at threshold depends on the distribution used for uncompensated conductances. Generated instances should thus exhibit similar activity to be considered degenerate ($\mathcal{D}_{\mathrm{generation}}$ concentrated), while maintaining large variability in conductance

values ($\mathcal{D}_{\text{generation}}$ spread). In essence, $\mathcal{D}_{\text{generation}}$ should maximize spread to facilitate degeneracy, yet remain concentrated enough to ensure similar activity within each population when DIC values are enforced.

Leak conductance distribution

In both distributions, we adopted a Gamma distribution for the leak conductance, rather than the uniform distribution used in [S6]. The Gamma distribution models variables with positive support and does not impose a hard upper bound. With suitable parameters, it also stabilizes normalization by minimizing density near zero. The shape and scale parameters are chosen to match the first and second moments of the corresponding uniform distribution from [S6]. The probability density function is:

$$X \sim \text{Gamma}(k, \theta) \implies p_X(x) = \frac{1}{\Gamma(k)\theta^k} x^{k-1} e^{-\frac{x}{\theta}} \quad , \tag{S13}$$

where k is the shape parameter and θ is the scale parameter.

Homogeneous scaling

In $\mathcal{D}_{\text{generation}}$, conductances are not independent: the leak conductance is sampled first, and the remaining conductances are scaled by the factor $\frac{g_{\text{leak}}}{g_{\text{leak,mean}}}$. This controls the effect of homogeneous scaling [S6], ensuring that relative conductance ratios are preserved across different leak values.

STG model distributions

Table S5 reports both distributions for the STG model. In $\mathcal{D}_{\text{generation}}$, no explicit bounds are set for $\bar{g}_{\text{Na}}$, $\bar{g}_{\text{Kd}}$, and $\bar{g}_{\text{H}}$, since these are compensated during the first step of the generation procedure (Algorithm S1).

Table S5. Distributions of maximal conductances in the STG model. Two distinct distributions are used at different stages: (a) $\mathcal{D}_{\text{analysis}}$, a broad distribution for preliminary analyses, and (b) $\mathcal{D}_{\text{generation}}$, used at inference and generation. Values are in mS cm^{-2}.

(a) Preliminary distribution $\mathcal{D}_{\text{analysis}}$ for broad exploratory analyses. Conductances are sampled independently from uniform distributions with predefined upper bounds, except for the leak conductance, which follows a gamma distribution.

Conductance $\bar{g}_i \sim \mathcal{U}(0; \bar{g}_{\text{max}})$	$\bar{g}_{\text{Na}}$	$\bar{g}_{\text{Kd}}$	$\bar{g}_{\text{CaT}}$	$\bar{g}_{\text{CaS}}$	$\bar{g}_{\text{KCa}}$	$\bar{g}_{\text{A}}$	$\bar{g}_{\text{H}}$
Maximum value $\bar{g}_{\text{max}}$	8000	350	12	50	250	600	0.7

Conductance $\bar{g}_i \sim \text{Gamma}(k, \theta)$	k	θ
g_{leak}	3	$\frac{1}{300}$

(b) Generation distribution $\mathcal{D}_{\text{generation}}$ for producing degenerate populations. This distribution is more concentrated and incorporates homogeneous scaling by normalizing conductances relative to the leak conductance.

Conductance $\bar{g}_i \sim \mathcal{U}(\bar{g}_{\text{min}}; \bar{g}_{\text{max}})\frac{g_{\text{leak}}}{g_{\text{leak,mean}}}$	$\bar{g}_{\text{Na}}$	$\bar{g}_{\text{Kd}}$	$\bar{g}_{\text{CaT}}$	$\bar{g}_{\text{CaS}}$	$\bar{g}_{\text{KCa}}$	$\bar{g}_{\text{A}}$	$\bar{g}_{\text{H}}$
Minimum value $\bar{g}_{\text{min}}$	—	70	2	6	140	—	—
Maximum value $\bar{g}_{\text{max}}$	—	140	7	22	180	—	—

Conductance $\bar{g}_i \sim \text{Gamma}(k, \theta)$	k	θ
g_{leak}	27	$\frac{1}{2570}$

DA model distributions

A similar approach was followed for the DA model, reported in Table S6. The only distinction is that $\bar{g}_{\mathrm{NMDA}}$ was set to be directly proportional to the leak conductance, corresponding to a fixed biological network around the cell across instances, as this current is mainly involved in synaptic transmission.

Table S6. Distributions of maximal conductances in the DA model. Two distinct distributions are used: (a) $\mathcal{D}_{\text{DA-analysis}}$ for preliminary analyses, and (b) $\mathcal{D}_{\text{DA-generation}}$ for inference and generation. Values are in $\mathrm{mS\,cm^{-2}}$.

(a) Preliminary distribution $\mathcal{D}_{\text{DA-analysis}}$ for exploratory analyses.

Conductance $\bar{g}_i \sim \mathcal{U}(0; \bar{g}_{\max})$	$\bar{g}_{\mathrm{Na}}$	$\bar{g}_{\mathrm{Kd}}$	$\bar{g}_{\mathrm{CaL}}$	$\bar{g}_{\mathrm{CaN}}$	$\bar{g}_{\mathrm{ERG}}$	$\bar{g}_{\mathrm{NMDA}}$
Maximum value $\bar{g}_{\max}$	60	20	0.1	0.12	0.25	0.012

Conductance $\bar{g}_i \sim \mathrm{Gamma}(k, \theta)$	k	θ
$\bar{g}_{\mathrm{leak}}$	3	$\frac{1}{300}$

(b) Generation distribution $\mathcal{D}_{\text{DA-generation}}$ for producing degenerate populations.

Conductance $\bar{g}_i \sim \mathcal{U}(\bar{g}_{\min}; \bar{g}_{\max}) \frac{g_{\mathrm{leak}}}{g_{\text{leak, mean}}}$	$\bar{g}_{\mathrm{Na}}$	$\bar{g}_{\mathrm{Kd}}$	$\bar{g}_{\mathrm{CaL}}$	$\bar{g}_{\mathrm{CaN}}$	$\bar{g}_{\mathrm{ERG}}$	$\bar{g}_{\mathrm{NMDA}}$
Minimum value $\bar{g}_{\min}$	—	6	0.015	—	—	0.012
Maximum value $\bar{g}_{\max}$	—	10	0.075	—	—	0.012

Conductance $\bar{g}_i \sim \mathrm{Gamma}(k, \theta)$	k	θ
g_{leak}	28.76	$\frac{1}{2238}$

C.5 Algorithmic procedure

We summarize the full procedure for generating populations of CBMs targeting specific DIC values as an algorithmic workflow (Algorithm S1). The method applies to a generic N-channel neuron model and consists of two main steps: generating a spontaneously active population and modulating it to reach a target point in the DIC space.

The first step produces a spontaneously active (spiking) population, while the second step iteratively adjusts the conductances to reach the target DIC values $(g_{\mathrm{s}}, g_{\mathrm{u}})$. We note that some negative conductances may remain in the DA model for $g_{\mathrm{u}} < 1.5$, but the regime-dependent selection of conductances for compensation reduces most invalid instances.

C.6 Synthetic dataset generation

We constructed a large open-source synthetic dataset spanning a broad range of DIC values and corresponding spike trains [S9]. Instead of sampling conductance parameters directly, we uniformly sampled the slow and ultra-slow DICs $g^* = (g_{\mathrm{s}}(V_{\mathrm{th}});\ g_{\mathrm{u}}(V_{\mathrm{th}}))$, since these components primarily shape firing activity. The fast DIC $g_{\mathrm{f}}(V_{\mathrm{th}})$ was constrained qualitatively to ensure spontaneous activity (sufficiently negative).

Sampling bounds

The bounds on the $(g_{\mathrm{s}}, g_{\mathrm{u}})$ space were derived by extensively sampling the $\mathcal{D}_{\mathrm{analysis}}$ distributions. Based on 4,000 instances (then 8,000 to ensure convergence) used to estimate the threshold voltage, we extracted the observed DIC bounds at threshold. For the STG model, the DIC values were

Algorithm S1 Iterative compensation procedure for CBM population generation.

Input: Number of channels N, DIC target $g_{\text{DICs}} = (g_{\text{s}}, g_{\text{u}})$, threshold value V_{th}, conductance distributions $\mathcal{D}_{\text{generation}}$ and the leak distribution Gamma.
Output: Population of CBM instances with compensated conductances.
procedure GenerateSpontaneousPopulation
    Draw leak conductance $g_{\text{leak}} \sim$ Gamma.
    Draw $N-3$ maximal conductances from $\mathcal{D}_{\text{generation}}$ scaled proportionally to g_{leak}.
    Compute the remaining 3 compensated conductances to impose a sufficiently negative $g_{\text{f}}(V_{\text{th}})$:
- STG: $\bar{g}_{\text{comp., spont.}} = (\bar{g}_{\text{Na}}, \bar{g}_{\text{Kd}}, \bar{g}_{\text{H}})$
- DA: $\bar{g}_{\text{comp., spont.}} = (\bar{g}_{\text{Na}}, \bar{g}_{\text{CaN}}, \bar{g}_{\text{ERG}})$

    Target the DIC values in Table S7a.
end procedure
procedure ModulatePopulationToTargetDIC(population, g_{DICs})
    Initialize iteration counter $k = 0$
    while $k < K_{\max}$ **do** ▷ e.g., $K_{\max} = 5$
        Update compensated conductances using the iterative solver based on $S(\bar{g}_{\text{comp.}})$
        to approach g_{DICs}.
        Increment $k \leftarrow k + 1$
    end while
    Remove any instances with negative compensated conductances.
    Select conductances to compensate as:

$$\bar{g}_{\text{comp.}} = \begin{cases} (\bar{g}_{\text{CaS}}, \bar{g}_{\text{H}}), & g_{\text{s}} < 0 \\ (\bar{g}_{\text{A}}, \bar{g}_{\text{H}}), & g_{\text{s}} > 0 \end{cases} \quad , \quad \bar{g}_{\text{DA-comp.}} = \begin{cases} (\bar{g}_{\text{ERG}}, \bar{g}_{\text{CaL}}), & g_{\text{s}} < 0 \\ (\bar{g}_{\text{ERG}}, \bar{g}_{\text{Kd}}), & g_{\text{s}} > 0 \end{cases} \tag{S14}$$

end procedure
Execute:
population $\leftarrow$ GenerateSpontaneousPopulation()
population $\leftarrow$ ModulatePopulationToTargetDIC(*population*, g_{DICs})
return *population*

largely enclosed in $(g_\mathrm{s}(V_\mathrm{th});\ g_\mathrm{u}(V_\mathrm{th})) \in [-20,\ 20] \times [-2,\ 20]$. We restricted the ultra-slow DIC to be positive (effectively using $[-20,\ 20] \times [0,\ 20]$) because we observed generation issues for negative values: populations were not degenerate, and such negative ultra-slow threshold DIC values corresponded to the negative outliers in Fig S2. We hypothesize that the shared threshold voltage approximation was not appropriate for such instances. For the DA model, the bounds were $[-15,\ 15] \times [0,\ 20]$, restricted during inference to $g_\mathrm{s} \in [-10,\ 15]$ as all instances generated from $g_\mathrm{s} < -10$ were silent.

Dataset construction

For each of the $N = 75{,}000$ sampled DIC pairs, we generated a degenerate population of $M = 16$ CBM instances using the iterative compensation algorithm, yielding a total of $|\mathcal{T}| = 1{,}200{,}000$ simulated neurons. Each instance was simulated under noisy current injection as described in Section A. The total simulation duration was 5000 ms for the STG model and 12 000 ms for the DA model. From each simulated trace, only spike times were retained:

$$V(t) \xrightarrow{\text{transformed into}} x = [t_1, t_2, \ldots, t_{N_\mathrm{spikes}}], \quad t_1 < t_2 < \cdots < t_{N_\mathrm{spikes}} \quad .$$

The final STG dataset consisted of 51.48% spiking and 48.28% bursting neurons, with 0.24% silent instances discarded. To prevent data leakage, instances from the same population were kept together and never split across partitions: $|\mathcal{T}_\mathrm{train}| = 1{,}000{,}000$ instances for training, $|\mathcal{T}_\mathrm{val}| = 200{,}000$ for validation, and 200,000 for testing. The test set was generated only after the architecture was fully trained and fixed, ensuring that performance metrics reflect generalization to unseen DIC targets and conductance configurations.

For the DA model, a reduced dataset of approximately 40% of the STG size was used, since transfer learning via LoRA adapters facilitates model adaptation with smaller datasets. The number of sampled configurations was set to yield approximately 25,000 active (non-silent) populations in total.

C.7 Target DIC values

Table S7 reports the exact DIC values targeted for the various results presented in this work.

The residual analysis of Fig S1 was based on sampling within the STG range reported above. We generated 5,000 populations of 250 instances from uniform sampling of the DIC targets and compensated $\bar{g}_\mathrm{CaS}$ and $\bar{g}_\mathrm{A}$. After discarding populations with negative conductance values, 1,633 remained for the analysis. The same random set of values was shared across all methods, so the analysis directly compared the results of the compensation step.

C.8 Spike definition and firing activity descriptors

Throughout the paper, we rely on descriptors of neuronal activity. These descriptors are **not** inputs to the deep learning architecture; however, they provide useful intermediates to characterize the firing pattern of individual instances (see Fig. S1 and Fig. 6 from the main text).

Spike extraction

When simulating CBMs, we obtained the full voltage trace $V(t)$. To simulate experimental conditions where only spike timing is recorded, we extracted spike times from $V(t)$ using a two-threshold method. A spike was considered to occur at the midpoint between the voltage crossing an upper threshold $V_\mathrm{up} = 10\,\mathrm{mV}$ from below and subsequently crossing a lower threshold

Table S7. Summary of target DIC values used in this work.

(a) Target DIC values at threshold for the first step of the generation procedure. These correspond to spontaneously active spiking populations.

Target DIC values	g_f	g_s	g_u
STG	-6.2	4	5
DA	-12.95	0.5	5

(b) Target DIC values for the example populations of Fig. S1

Target DIC values	g_s	g_u
Spiking	5	4
Bursting	-2.71	5.63

$V_\mathrm{down} = 0\,\mathrm{mV}$ from above. Formally, for each spike i:

$$t_i = \frac{t_\mathrm{up}^{(i)} + t_\mathrm{down}^{(i)}}{2} \quad ,$$

where $t_\mathrm{up}^{(i)}$ is the time when $V(t)$ first exceeds V_up and $t_\mathrm{down}^{(i)}$ is the time when it next falls below V_down. Applying this procedure to all spikes produces the sequence:

$$V(t) \xrightarrow{\text{spike extraction}} x = [t_1, t_2, \ldots, t_{N_\mathrm{spikes}}] \quad .$$

Activity descriptors

Given a sequence of spike times $x = [t_1, \ldots, t_{N_\mathrm{spikes}}]$ and the corresponding inter-spike intervals $x_\mathrm{ISI} = \Delta x = [t_2 - t_1, t_3 - t_2, \ldots, t_{N_\mathrm{spikes}} - t_{N_\mathrm{spikes}-1}]$, we computed the following descriptors.

Spiking activity. We reported the mean firing frequency:

$$f_\mathrm{spk} = \left(\frac{1}{N_\mathrm{spikes} - 1} \sum_{i=1}^{N_\mathrm{spikes}-1} \Delta x_i \right)^{-1} \quad . \tag{S15}$$

Bursting activity. We computed metrics based on burst extraction. From the spike time sequence, we constructed a sequence of bursts $\mathcal{B} = [B_1, \ldots, B_{N_\mathrm{burst}}]$, where each B_i is a sequence of $N_{i,\mathrm{spikes}}$ consecutive spikes, split based on the mid-range value of x_ISI. The first spike of each burst was such that the duration since the previous spike exceeded $\frac{\min(x_\mathrm{ISI}) + \max(x_\mathrm{ISI})}{2}$. The first and last bursts were discarded, as they could be incomplete due to simulation boundaries. We then reported:

1. The mean intra-burst frequency:

$$f_\mathrm{intra} = \left(\frac{1}{N_\mathrm{burst}} \sum_{i=1}^{N_{i,\mathrm{spikes}}-1} \Delta B_i \right)^{-1} \quad . \tag{S16}$$

2. The mean inter-burst frequency, i.e., the inverse of the mean duration between bursts:

$$f_\mathrm{inter} = \left(\frac{1}{N_\mathrm{burst} - 1} \sum_{i=1}^{N_\mathrm{burst}-1} (B_{i+1})_{N_{i+1,\mathrm{spikes}}} - (B_i)_1 \right)^{-1} \quad . \tag{S17}$$

3. The mean burst duration:

$$\text{Duration} = \frac{1}{N_{\text{burst}}} \sum_{i=1}^{N_{\text{burst}}} \sum_{j=1}^{N_{i,\text{spikes}}-1} (\Delta B_i)_j \quad . \tag{S18}$$

4. The mean number of spikes per burst:

$$\# = \frac{1}{N_{\text{burst}}} \sum_{i=1}^{N_{\text{burst}}} N_{i,\text{spikes}} \quad . \tag{S19}$$

   Some bursting neurons were slightly irregular (for example, producing alternating bursts of 3 and 4 spikes), and we kept the mean as a non-integer value in those cases. This is why this metric can take non-integer values despite the quantized nature of spikes per burst.

D Heterogeneous populations at the spiking-bursting transition

Throughout the paper, figures such as Fig. 2 from the main text show an overlap between the DIC region associated with bursting and the one associated with spiking. The transition between regimes is roughly located at $g_{\text{s}} \approx 0$, with $g_{\text{s}} > 0$ corresponding to spiking and $g_{\text{s}} < 0$ to bursting, and almost no dependency on g_{u}. However, in practice, this transition is not sharp, and a whole region marks it. In this region, generated populations are heterogeneous, containing a mix of spiking and bursting instances. The bursting instances in this transition zone typically produce doublets (bursts with only 2 spikes) or alternations of single spikes and doublets.

To characterize this region, we generated populations across the DIC space and computed the class entropy of each population P:

$$\mathcal{H}(P) = - \sum_{t \in \{\text{silent},\text{spiking},\text{bursting}\}} p_t \log_3(p_t) \in [0, 1] \quad , \tag{S20}$$

where homogeneous populations have zero entropy and highly heterogeneous populations have an entropy of one. Figure S3 highlights the spiking-bursting transition based on this entropy measure. A small region of mixed spiking-silent instances is also visible in the bottom right of the DIC space.

This analysis directly addresses the question of what proportion of generated models preserve the nominal activity type. Outside the narrow transition zone near $g_{\text{s}} \approx 0$, populations are homogeneous (entropy ≈ 0), meaning that virtually all instances preserve the expected activity type: spiking for $g_{\text{s}} > 0$ and bursting for $g_{\text{s}} < 0$. The mixed populations observed at the transition are not a failure of the method but rather reflect a genuine dynamical property: near the bifurcation between regimes, the system is sensitive to small parameter variations, and individual instances within a degenerate population may fall on either side of the boundary. Silent neurons are rare throughout the DIC space, representing less than 0.24% of all generated instances, and occur primarily at the extreme boundaries where DIC constraints approach non-physiological values.

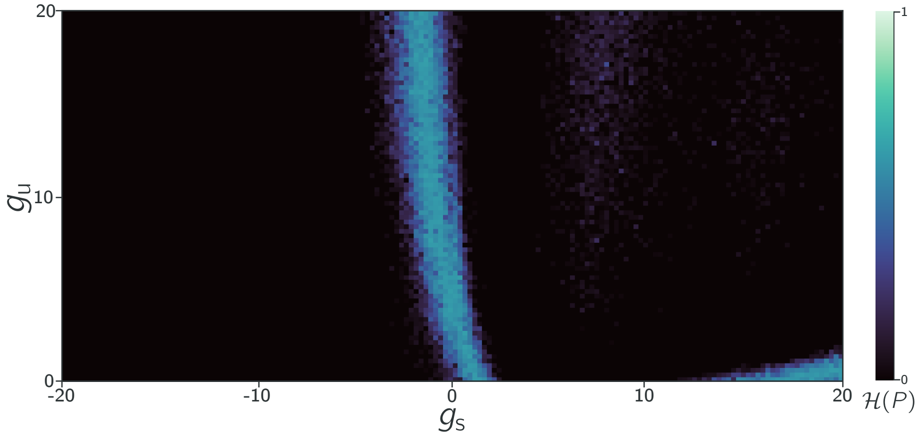

Fig S3. Population heterogeneity across the STG DIC space. The spiking-bursting transition ($g_\mathrm{s} \approx 0$) corresponds to a highly heterogeneous region where populations contain a mix of spiking and bursting instances.

E Deep learning architecture and training

E.1 Architecture overview

The architecture (Fig S4) comprises an attention-based encoder [S10] and a multi-headed decoder. The encoder maps variable-length spike time sequences to a fixed-size latent vector $z_\mathrm{latent} \in \mathbb{R}^{d_\mathrm{latent}}$, and the decoder processes this representation through three parallel heads to produce both the primary output and auxiliary predictions that regularize training.

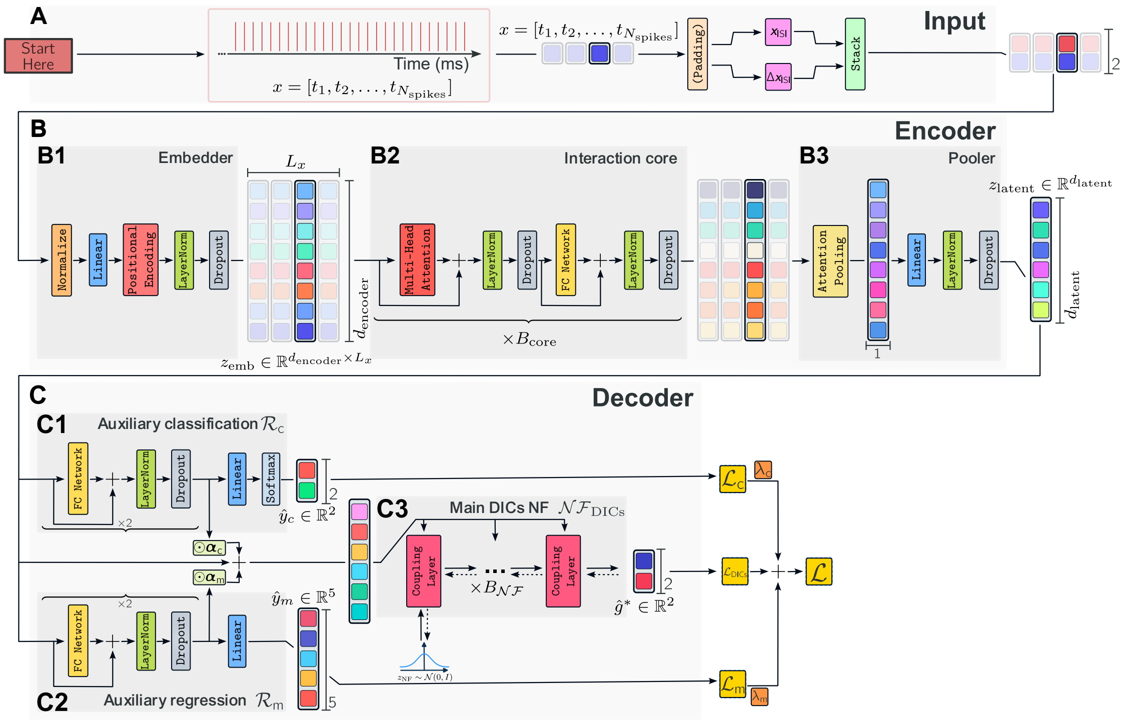

Fig S4. The deep learning architecture. (A) The input to the model consists of spike time sequences, from which ISIs and delta ISIs are extracted. These features are then stacked and fed into the encoder. **(B)** The encoder processes the input through three main components: the embedder, the interaction core, and the pooler. The embedder transforms the input sequence into a normalized, higher-dimensional representation. The interaction core processes this representation using multi-head attention mechanisms and fully-connected networks. The pooler aggregates the variable-length representation into a fixed-size latent representation. **(C)** The decoder transforms the fixed-size latent representation into a conditional density over DICs via a normalizing flow, and includes auxiliary heads for classifying neuronal activity and regressing electrical activity metrics.

Encoder. Given a spike train x, we compute inter-spike intervals (ISIs) and their second-order differences to enhance burst detection. A logarithmic transform $\log(1 + x_{\text{ISI}})$ is applied to stabilize the ISI distribution, and the resulting features are standardized using training set statistics. The embedder (Fig S4B1) projects these normalized features into a higher-dimensional space enriched with sinusoidal positional encoding [S10]. The interaction core (Fig S4B2) consists of B_{core} stacked transformer blocks, each combining multi-head self-attention with a position-wise fully connected network using GELU activations [S11], residual connections, layer normalization [S12], and dropout. A self-attention pooling layer (Fig S4B3) aggregates the variable-length output into a fixed-size latent vector $z_{\text{latent}} \in \mathbb{R}^{d_{\text{latent}}}$.

Decoder. The decoder comprises three heads operating on the shared latent representation. The primary head is a RealNVP-style normalizing flow [S13] ($\mathcal{NF}_{\text{DICs}}$, Fig S4C3) that models the conditional density $p_\theta(g^* \mid z_{\text{latent}})$ over DIC targets using stacked affine coupling layers, each conditioned on the latent vector. Two auxiliary heads are used only during training: a classification head ($\mathcal{R}_c$, Fig S4C1) that predicts the firing regime (spiking or bursting), and a regression head ($\mathcal{R}_m$, Fig S4C2) that predicts five activity descriptors (mean firing rate for spiking; intra-burst frequency, inter-burst frequency, burst duration, and spikes per burst for bursting). The auxiliary latent outputs are integrated with the encoder output via learnable element-wise mixing before conditioning the normalizing flow. At inference time, only the normalizing flow head is used.

E.2 Training procedure

The model is trained end-to-end by minimizing a composite loss $\mathcal{L} = \mathcal{L}_{\text{flow}} + \lambda_{\text{m}}\mathcal{L}_{\text{m}} + \lambda_{\text{c}}\mathcal{L}_{\text{c}}$, combining the negative log-likelihood of the normalizing flow ($\mathcal{L}_{\text{flow}}$) with a masked mean squared error on the activity descriptors ($\mathcal{L}_{\text{m}}$, where the mask selects regime-appropriate metrics) and a balanced cross-entropy for firing regime classification ($\mathcal{L}_{\text{c}}$). The weights λ_{m} and λ_{c} control the relative importance of the auxiliary tasks.

Three forms of data augmentation are applied during training: random cropping of spike trains to a window $D \sim \mathcal{U}[N_{\text{spikes}}/2, N_{\text{spikes}}]$, Gaussian jitter $\epsilon_i \sim \mathcal{N}(0, (2\,\text{ms})^2)$ on spike times, and 5% spike dropout. These are applied independently per sample at each update.

Hyperparameters were optimized via random search over 100 configurations [S14], using a lightweight pointwise regression decoder in place of the normalizing flow to reduce computational cost. Table S8 lists the explored hyperparameters and their distributions, and Table S9 reports the selected values. The selected configuration was then used to train the full model with the normalizing flow head. We used AdamW [S15] with a cosine annealing schedule with warm restarts (period $T_i = 10$ epochs, minimum learning rate $\eta_{\min} = \eta/10$). Training ran for 200 epochs, with validation every quarter epoch; the checkpoint with the lowest validation loss $\mathcal{L}_{\text{flow}}$ was retained. The final architecture comprises 150,572 trainable parameters.

Table S8. Hyperparameters and distributions explored in random search. $\tilde{\lambda}_{\text{c}}$ and $\tilde{\lambda}_{\text{m}}$ are relative weights of the auxiliary losses to the primary DICs loss.

**Hyperparameter**	**Type / Distribution**	**Values or Range**
Learning rate (η)	Log-uniform	$[10^{-5}, 5 \cdot 10^{-3}]$
Dropout (p_{dropout})	Uniform	$[0.0, 0.4]$
Latent space dimension (d_{latent})	Discrete	$\{16, 32, 64, 128\}$
Encoder space dimension (d_{encoder})	Discrete	$\{16, 32, 64, 128\}$
Number of heads (H)	Discrete	$\{2, 4, 8\}$
Number of encoder blocks (B_{core})	Discrete	$\{1, 2, 3, 4, 5, 6, 7, 8\}$
Number of decoder blocks ($B_{\mathcal{R}_{\text{DICs}}}$)	Discrete	$\{2, 4, 6, 8, 10\}$
Activation function ($\sigma(\cdot)$)	Categorical	`relu`, `gelu`, `silu`, `tanh`
Apply log transform to input?	Boolean	`true`, `false`
Regression weighting factor ($\tilde{\lambda}_{\text{m}}$)	Uniform	$[0.01, 10.0]$
Classification weighting factor ($\tilde{\lambda}_{\text{c}}$)	Uniform	$[0.01, 10.0]$
Batch size ($\lvert B \rvert$)	Discrete	$\{16, 32, 64, 128\}$

E.3 Evaluation metrics

During hyperparameter optimization, the primary selection metric was the MAE of the pointwise regression decoder on the validation set. At test time, we report auxiliary task performance: MAE on activity descriptors for the regression head, and balanced accuracy for the classification head. These metrics verify that the encoder captures meaningful temporal structure from raw spike trains. The quality of predicted DIC targets is ultimately assessed through the end-to-end posterior predictive checks described in the main text (Fig. 4 and Fig. 5).

E.4 Posterior calibration diagnostics

Because our architecture learns a conditional density $p_\theta(g^* \mid x)$ rather than a point estimate, it is essential to verify that the learned posteriors are well calibrated. Following the recommendations of [S16], we assessed calibration using three complementary diagnostics: Tests of Accuracy with

Table S9. Final hyperparameters for the STG backbone. Values selected after random search, yielding the best validation performance.

Hyperparameter	Final value
Learning rate (η)	2.10×10^{-5}
Dropout (p_{dropout})	0.034
Latent space dimension (d_{latent})	16
Encoder space dimension (d_{encoder})	64
Number of heads (H)	8
Number of encoder blocks (B_{core})	4
Number of decoder blocks ($B_{\mathcal{R}_{\text{DICs}}}$)	2
Activation function ($\sigma(\cdot)$)	`gelu`
Apply log transform to input?	`true`
Regression weighting factor ($\tilde{\lambda}_{\text{m}}$)	0.0919
Classification weighting factor ($\tilde{\lambda}_{\text{c}}$)	5.44
Batch size ($\lvert B \rvert$)	32

Random Points (TARP) [S17], simulation-based calibration (SBC) rank histograms [S18], and expected coverage tests [S19]. All diagnostics were evaluated on held-out validation data for both the STG and DA models. Results are summarized in Fig S5.

Tests of Accuracy with Random Points (TARP)

TARP provides a joint calibration diagnostic sensitive to both coverage and bias. For each of N validation inputs, $L = 2{,}000$ posterior samples are drawn from the learned conditional density. A set of $R = 1{,}000$ random reference points is sampled uniformly over the DIC parameter space. For each reference point, the test computes the fraction of posterior samples that lie closer (in Euclidean distance) to the reference than the true parameter value does. If the posterior is well calibrated, these fractions should be uniformly distributed and the resulting empirical coverage curve should closely follow the identity line. Deviations above the diagonal indicate conservative (overdispersed) posteriors, while deviations below indicate overconfident ones. We quantified departure from ideal calibration using the expected calibration error (ECE), defined as the mean absolute deviation between the empirical coverage curve and the diagonal. 95 % bootstrap confidence intervals were computed from 1,000 bootstrap resamples of the reference points.

For both models, the empirical TARP coverage curves closely follow the diagonal and remain within the 95 % confidence band across all credibility levels (Fig S5, first column). The ECE values are small for both models, indicating no detectable joint miscalibration.

Simulation-based calibration rank histograms

SBC provides a marginal calibration check for each parameter dimension independently [S18]. For each validation input, the rank of the true DIC value among the L posterior samples is computed: $r = \sum_{l=1}^{L} \mathbb{I}[g_l < g^*]$. If the posterior is calibrated, the distribution of ranks should be uniform. A U-shaped histogram signals an overdispersed posterior, an inverted-U shape signals overconcentration, and a skewed histogram signals systematic bias. We assessed uniformity using a chi-squared goodness-of-fit test with 20 histogram bins.

For both models and both DIC dimensions, the rank distributions are approximately uniform and the chi-squared tests do not reject uniformity at the 5 % significance level (Fig S5, second and third columns), confirming that the marginal posteriors are well calibrated.

Expected coverage tests

As a third diagnostic, we performed marginal expected coverage tests [S19]. For each credibility level $\alpha \in [0, 1]$, the symmetric α-credible interval is constructed from the posterior samples for each validation input, and the empirical coverage is computed as the fraction of inputs for which the true value falls within this interval. We summarized calibration quality using the ECE and computed 95 % bootstrap confidence bands from 300 resamples.

For both models and both dimensions, the empirical coverage closely tracks the diagonal and falls within the confidence band (Fig S5, fourth and fifth columns). The empirical coverage of the 90 % credible interval is close to the nominal level in all cases, confirming the absence of systematic over- or under-coverage.

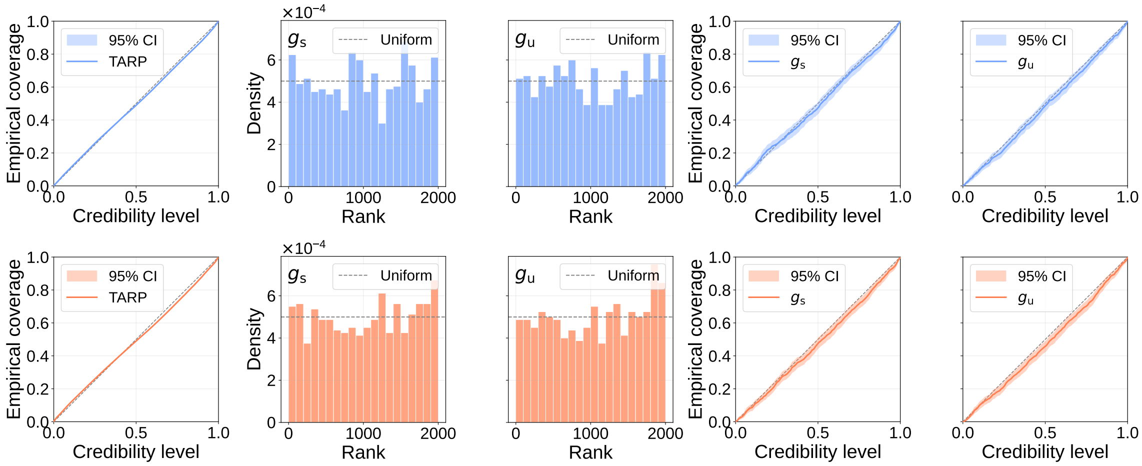

Fig S5. Posterior calibration diagnostics for the STG (top, blue) and DA (bottom, orange) models. From left to right: TARP coverage curves with 95 % bootstrap confidence bands; SBC rank histograms for $g_s(V_{th})$ and $g_u(V_{th})$ with the expected uniform density (dashed); and marginal expected coverage curves for $g_s(V_{th})$ and $g_u(V_{th})$ with 95 % bootstrap confidence bands. *STG:* TARP ECE = 0.0104; SBC χ^2 $p = 0.060$ (g_s), 0.493 (g_u); expected coverage ECE = 0.0150 (g_s), 0.0181 (g_u); 90 % credible interval coverage = 0.874 (g_s), 0.884 (g_u). *DA:* TARP ECE = 0.0140; SBC χ^2 $p = 0.522$ (g_s), 0.178 (g_u); expected coverage ECE = 0.0227 (g_s), 0.0286 (g_u); 90 % credible interval coverage = 0.875 (g_s), 0.885 (g_u). SBC χ^2 tests do not reject the null hypothesis of uniform ranks at the 5 % significance level for either model or parameter; TARP and marginal expected coverage ECE values are small, indicating no meaningful deviation from calibration.

Taken together, the three diagnostics reveal no evidence of posterior miscalibration for either model. These results provide strong evidence that the learned conditional density captures the structure of the posterior over DIC values, including its degeneracy, in a statistically reliable manner.

E.5 Low-Rank Adaptation and transfer to the DA model

To adapt the pipeline to the DA neuron model, we applied parameter-efficient fine-tuning using Low-Rank Adaptation (LoRA) [S20]. LoRA adapters were introduced in the fully connected layers of the network, while attention layers remained unmodified (Fig S6A). The majority of backbone parameters were frozen, and only the newly introduced LoRA parameters were updated during training. The input normalization layer was recalculated based on the DA training set.

LoRA adapters rely on low-rank parameterized matrices $A \in \mathbb{R}^{d\times r}$ and $B \in \mathbb{R}^{r\times k}$ (Fig S6B), which modify each weight matrix $W \in \mathbb{R}^{d\times k}$ as:

$$Wx \xrightarrow[\text{(red circle)}]{\text{LoRA adapter}} \underbrace{Wx}_{\text{frozen (blue circle)}} + \underbrace{\frac{1}{r}xAB}_{\text{learnable (red circle)}} . \tag{S21}$$

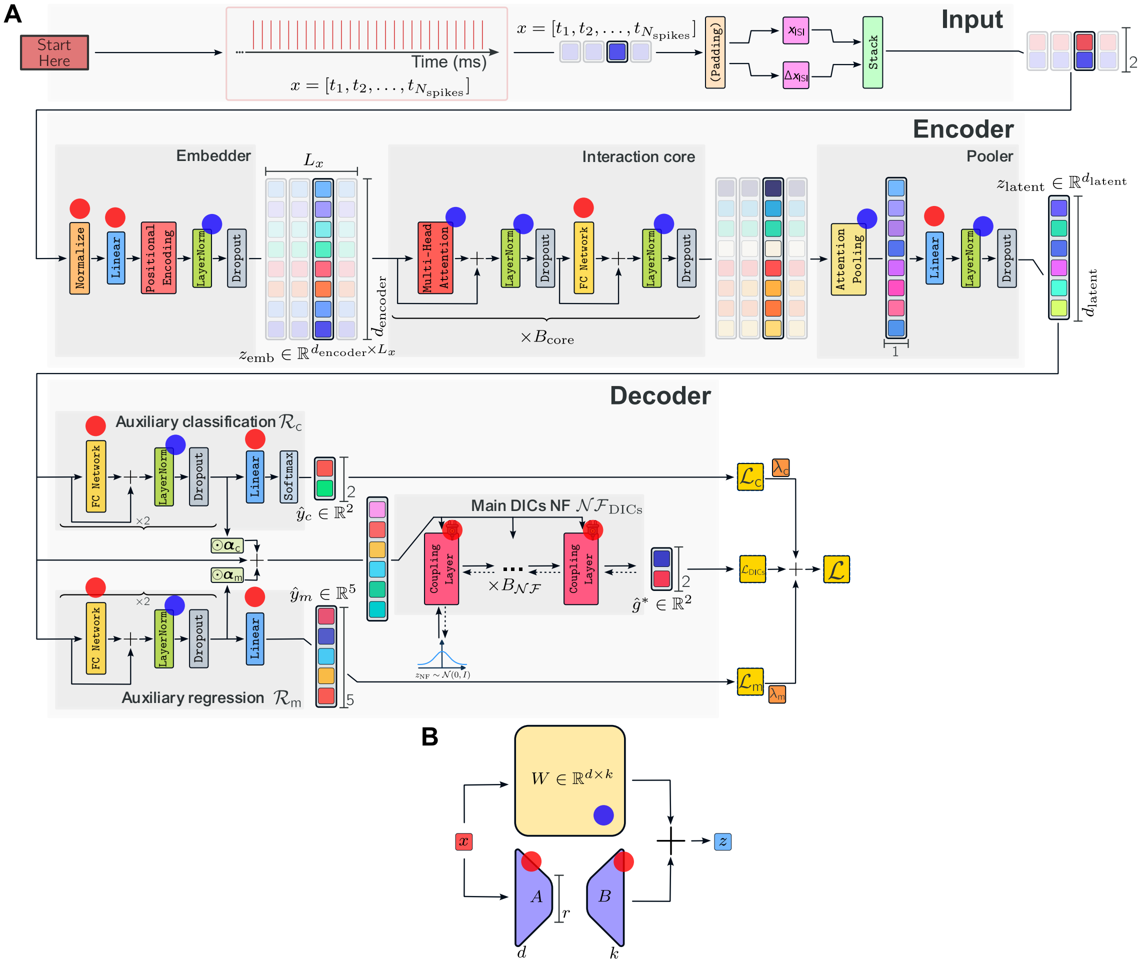

Fig S6. The deep learning architecture with LoRA to transfer from the STG to the DA. (A) The red circle indicates the insertion of a LoRA adapter, where the original layer parameters are frozen (blue circle) and a new linear projection is added in an additive manner with new learnable parameters (panel (B)). The blue circle signifies that the parameters of the corresponding layer are not retrained, preserving its original functionality and weights. The only exception is the normalization block (orange) at pipeline input, which is directly replaced by the new normalization statistics calculated on the DA train set. **(B)** The LoRA adapter introduces a small number of additional parameters through low-rank matrices $A \in \mathbb{R}^{d\times r}$ and $B \in \mathbb{R}^{r\times k}$. These matrices modify the matrix multiplication in fully connected layers by adding a learnable component to the frozen pre-trained weights $W \in \mathbb{R}^{d\times k}$.

We set the rank to $r = 32$, which yields satisfactory transfer performance. This introduces additional parameters representing approximately 40% of the total parameters that would be required for training a model from scratch. While the backbone is already lightweight (150,572 parameters), this result serves as a proof of concept that LoRA provides a practical strategy for

extending the pipeline to new conductance-based models. In settings where the framework must support many distinct CBMs, each new model would only require storing a small set of LoRA weights on top of a shared backbone, making the approach well suited for scaling within a single unified framework.

The DA dataset was generated using the same procedure as for the STG model (Section C.6). Due to a substantial fraction of silent populations in the DA parameter space, these were discarded, and the number of sampled configurations was set to yield approximately 25,000 active populations. The same training protocol was used, with the LoRA-adapted model trained on this reduced dataset. The smaller dataset size is sufficient because LoRA leverages representations already learned by the STG backbone, requiring fewer examples to adapt to the new model.

F Pipeline applied to sparse, excitable-like spike trains

The training dataset samples DIC values within the spontaneously active region of parameter space, where $g_{\mathrm{f}}(V_{\mathrm{th}})$ is negative (see Section C.6), but the architecture itself makes no assumption of spontaneity. To illustrate this point, we applied the trained pipeline, without any retraining, to two sparse spike trains generated by injecting, on top of the standard stochastic input, a constant hyperpolarizing current set just below the rheobase of an otherwise spontaneously active STG neuron, where the rheobase was estimated from a slow ramp protocol. This suppresses the limit cycle and shifts the neuron into an excitable-like regime in which spikes occur only when noise excursions transiently cross threshold.

The resulting inputs lie outside the training distribution. Each input spike train was passed through the full pipeline, and the same hyperpolarizing current was preserved when simulating the inferred population. Since the inferred neurons remain in the spontaneous region of DIC space, their input resistance differs from that of the input neuron, so the preserved holding current no longer sits just below the rheobase of the output neurons. This rheobase mismatch is intrinsic to the proof-of-concept setting, since matching the input resistance would itself require the targeted retraining outlined in the main text Discussion. We therefore expect the firing mode of input and output to match, while the exact firing statistics, in particular spike sparsity, may differ.

Figure S7 confirms this expectation: for both a bursting-like and a spiking-like sparse input, the inferred population correctly recovers the firing mode, while the output traces are denser than the inputs, consistent with the rheobase argument above. These observations support the claim that the qualitative spiking/bursting classification carries through to inputs the pipeline has never encountered during training, and that quantitatively matching the statistics of excitable-like recordings would require enlarging the training distribution to span $g_{\mathrm{f}}(V_{\mathrm{th}}) > 0$, adding $g_{\mathrm{f}}(V_{\mathrm{th}})$ as a third inference target alongside $g_{\mathrm{s}}(V_{\mathrm{th}})$ and $g_{\mathrm{u}}(V_{\mathrm{th}})$, and extending the compensation step to enforce all three DIC components.

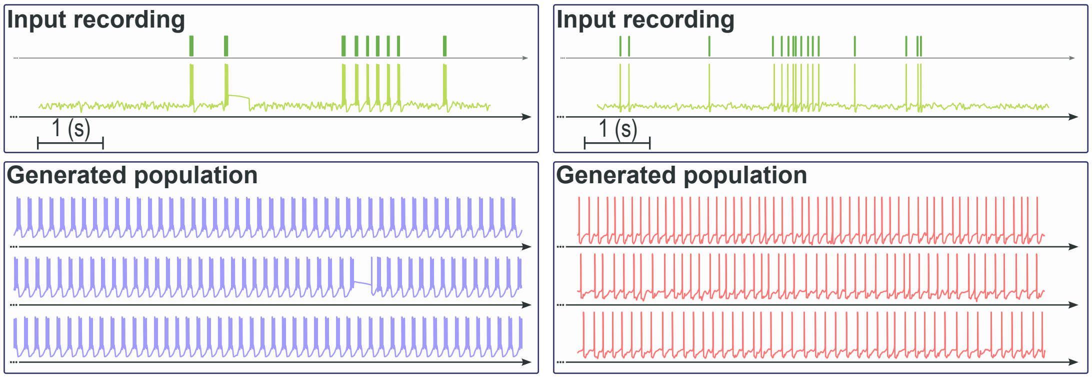

Fig S7. Pipeline applied to sparse, excitable-like spike trains generated by sub-rheobase hyperpolarizing current injection. For each firing mode (left: bursting-like, right: spiking-like), the top panel shows the input spike train obtained by injecting a constant hyperpolarizing current set just below the rheobase of the input neuron, on top of the standard stochastic input. The three lower panels show representative output traces from the inferred population, simulated with the same hyperpolarizing current preserved. These inputs lie entirely outside the training distribution, since the pipeline was trained on spontaneously active samples ($g_{\mathrm{f}}(V_{\mathrm{th}}) < 0$).

References

S1. Liu Z, Golowasch J, Marder E, Abbott LF. A model neuron with activity-dependent conductances regulated by multiple calcium sensors. J Neurosci. 1998;18(7):2309-20. doi:10.1523/JNEUROSCI.18-07-02309.1998.

S2. Qian K, Yu N, Tucker KR, Levitan ES, Canavier CC. Mathematical analysis of depolarization block mediated by slow inactivation of fast sodium channels in midbrain dopamine neurons. J Neurophysiol. 2014;112(11):2779-90. doi:10.1152/jn.00578.2014.

S3. Virtanen P, Gommers R, Oliphant TE, Haberland M, Reddy T, Cournapeau D, et al. SciPy 1.0: fundamental algorithms for scientific computing in Python. Nat Methods. 2020;17:261-72. doi:10.1038/s41592-019-0686-2.

S4. Shampine LF, Reichelt MW. The MATLAB ODE suite. SIAM J Sci Comput. 1997;18(1):1-22. doi:10.1137/S1064827594276424.

S5. Drion G, Franci A, Dethier J, Sepulchre R. Dynamic input conductances shape neuronal spiking. eNeuro. 2015;2(1). doi:10.1523/ENEURO.0031-14.2015.

S6. Fyon A, Franci A, Sacré P, Drion G. Dimensionality reduction of neuronal degeneracy reveals two interfering physiological mechanisms. PNAS Nexus. 2024;3(10):pgae415. doi:10.1093/pnasnexus/pgae415.

S7. Fyon A, Sacré P, Franci A, Drion G. Reliable neuromodulation from adaptive control of ion channel expression. IFAC-PapersOnLine. 2023;56(2):458-63. doi:10.1016/j.ifacol.2023.10.1610.

S8. Goaillard JM, Marder E. Ion channel degeneracy, variability, and covariation in neuron and

circuit resilience. Annu Rev Neurosci. 2021;44:335-57. doi:10.1146/annurev-neuro-092920-121538.

S9. Brandoit J, Ernst D, Drion G, Fyon A. Spike-train datasets from conductance-based neuron models; 2025. Dataset. Available from: Zenodo. doi:10.5281/zenodo.16912160.

S10. Vaswani A, Shazeer N, Parmar N, Uszkoreit J, Jones L, Gomez AN, et al. Attention is all you need. In: Proceedings of the 31st International Conference on Neural Information Processing Systems; 2017. p. 6000-10. doi:10.48550/arXiv.1706.03762.

S11. Hendrycks D, Gimpel K. Gaussian error linear units (GELUs); 2023. Preprint. Available from: https://arxiv.org/abs/1606.08415. doi:10.48550/arXiv.1606.08415.

S12. Ba JL, Kiros JR, Hinton GE. Layer normalization; 2016. Preprint. Available from: https://arxiv.org/abs/1607.06450. doi:10.48550/arXiv.1607.06450.

S13. Dinh L, Sohl-Dickstein J, Bengio S. Density estimation using Real NVP; 2017. Preprint. Available from: https://arxiv.org/abs/1605.08803. doi:10.48550/arXiv.1605.08803.

S14. Bergstra J, Bengio Y. Random search for hyper-parameter optimization. J Mach Learn Res. 2012;13:281-305.

S15. Loshchilov I, Hutter F. Decoupled weight decay regularization; 2019. Preprint. Available from: https://arxiv.org/abs/1711.05101. doi:10.48550/arXiv.1711.05101.

S16. Hermans J, Delaunoy A, Rozet F, Wehenkel A, Begy V, Louppe G. A trust crisis in simulation-based inference? Your posterior approximations can be unfaithful; 2022. Preprint. Available from: https://arxiv.org/abs/2110.06581. doi:10.48550/arXiv.2110.06581.

S17. Lemos P, Coogan A, Hezaveh Y, Perreault-Levasseur L. Sampling-based accuracy testing of posteriors in general-purpose inference engines. In: Proceedings of the 40th International Conference on Machine Learning; 2023. .

S18. Talts S, Betancourt M, Simpson D, Vehtari A, Gelman A. Validating Bayesian inference algorithms with simulation-based calibration; 2018. Preprint. Available from: https://arxiv.org/abs/1804.06788. doi:10.48550/arXiv.1804.06788.

S19. Cook SR, Gelman A, Rubin DB. Validation of software for Bayesian models using posterior quantiles. J Comput Graph Stat. 2006;15(3):675-92. doi:10.1198/106186006X136976.

S20. Hu EJ, Shen Y, Wallis P, Allen-Zhu Z, Li Y, Wang S, et al.. LoRA: low-rank adaptation of large language models; 2021. Preprint. Available from: https://arxiv.org/abs/2106.09685. doi:10.48550/arXiv.2106.09685.
